# Democratizing cheminformatics: interpretable chemical grouping using an automated KNIME workflow

**DOI:** 10.1186/s13321-024-00894-1

**Published:** 2024-08-16

**Authors:** José T. Moreira-Filho, Dhruv Ranganath, Mike Conway, Charles Schmitt, Nicole Kleinstreuer, Kamel Mansouri

**Affiliations:** 1 National Toxicology Program Interagency Center for the Evaluation of Alternative Toxicological Methods, Division of Translational Toxicology, National Institute of Environmental Health Sciences, Research Triangle Park, North Carolina USA; 2 University of North Carolina at Chapel Hill, Chapel Hill, North Carolina USA; 3 National Institute of Environmental Health Sciences, Research Triangle Park, North Carolina USA; 4 Division of Translational Toxicology, National Institute of Environmental Health Sciences, Research Triangle Park, North Carolina USA

**Keywords:** Chemical grouping, KNIME workflow, Machine learning, Explainable artificial intelligence, SHapley additive exPlanations, Feature selection, Data visualization, Unsupervised clustering, Supervised classification

## Abstract

With the increased availability of chemical data in public databases, innovative techniques and algorithms have emerged for the analysis, exploration, visualization, and extraction of information from these data. One such technique is chemical grouping, where chemicals with common characteristics are categorized into distinct groups based on physicochemical properties, use, biological activity, or a combination. However, existing tools for chemical grouping often require specialized programming skills or the use of commercial software packages. To address these challenges, we developed a user-friendly chemical grouping workflow implemented in KNIME, a free, open-source, low/no-code, data analytics platform. The workflow serves as an all-encompassing tool, expertly incorporating a range of processes such as molecular descriptor calculation, feature selection, dimensionality reduction, hyperparameter search, and supervised and unsupervised machine learning methods, enabling effective chemical grouping and visualization of results. Furthermore, we implemented tools for interpretation, identifying key molecular descriptors for the chemical groups, and using natural language summaries to clarify the rationale behind these groupings. The workflow was designed to run seamlessly in both the KNIME local desktop version and KNIME Server WebPortal as a web application. It incorporates interactive interfaces and guides to assist users in a step-by-step manner. We demonstrate the utility of this workflow through a case study using an eye irritation and corrosion dataset.

**Scientific contributions**

This work presents a novel, comprehensive chemical grouping workflow in KNIME, enhancing accessibility by integrating a user-friendly graphical interface that eliminates the need for extensive programming skills. This workflow uniquely combines several features such as automated molecular descriptor calculation, feature selection, dimensionality reduction, and machine learning algorithms (both supervised and unsupervised), with hyperparameter optimization to refine chemical grouping accuracy. Moreover, we have introduced an innovative interpretative step and natural language summaries to elucidate the underlying reasons for chemical groupings, significantly advancing the usability of the tool and interpretability of the results.

**Supplementary Information:**

The online version contains supplementary material available at 10.1186/s13321-024-00894-1.

## Introduction

Over recent decades, advances in experimental methodologies have generated substantial bioactivity data for chemicals. Several data-sharing initiatives made a significant portion of this data publicly accessible through digital databases [1–5]. Additionally, databases of computationally generated chemical structures and extensive catalogs were offered by chemical vendors [6–8]. This surge in data availability has spurred the development of methods and tools for processing, analyzing, and modeling chemical data, aiding drug discovery and toxicity assessment [9–11].

Chemical grouping, including clustering and classification, categorizes compounds based on shared characteristics into distinct groups. This process relies on the concept of similarity, where compounds within a group are more alike than those in different groups. Compounds can be grouped based on molecular descriptors, substructures, physicochemical properties, use categories, or biological activities. The rationale is that high similarity indicates similar properties or activities [12–14]. Chemical grouping serves various purposes, including assessing diversity, understanding mechanisms of action, extracting structure–activity relationships (SARs), conducting safety and risk assessments through read-across approaches, and prioritizing chemicals for testing [15–20].

Chemical grouping utilizes advanced clustering and classification algorithms to uncover underlying patterns in datasets [21]. However, interpreting how these algorithms group data points can be challenging [22, 23]. Explainable artificial intelligence (XAI) strategies, [24] such as SHapley Additive exPlanations (SHAP) [25], aim to enhance interpretability. SHAP can identify influential features in grouping decisions, providing transparency to machine learning (ML) models [26–28].

Enhancing openness and sharing of chemical data and modeling methods is crucial to broaden accessibility [29–31]. However, open-source cheminformatics tools often lack documentation and require significant programming skills for setup and use [32, 33]. Democratizing these tools to all levels of expertise demands easy-to-install, intuitive user-friendly graphical user interfaces (GUIs) with well-documented guidance [34–37]. Yet, creating such interfaces also requires proficiency in multiple programming languages such as Python, R, Java, HTML, and JavaScript.

Low-code or no-code platforms offer a solution to the demand for advanced programming skills, allowing for the development and deployment of applications with little to no coding. Users select, arrange, configure and connect components from standard libraries and third-party plugins to develop applications through a visual programming paradigm within a GUI. These platforms empower domain experts, including those without programming backgrounds, to collaborate in the development process, enhancing application quality [38–43]. Besides improving efficiency and reducing costs, they accelerate development rates by five to ten times compared to traditional hand coding, potentially leading to 70% of enterprise applications being created using low-code solutions by 2025 [40, 42].

Konstanz Information Miner (KNIME) is a free and open-source low/no-code data analytics platform with a broad range of capabilities and a thriving cheminformatics and bioinformatics community. Its modular setup enables users to visually assemble and modify analysis flows using standardized building blocks called nodes. Nodes are connected by pipes that transfer data and instructions, forming the data processing workflows [44, 45].

Introducing the Modeling and Visualization Pipeline (MoVIZ), a user-friendly tool developed on the KNIME platform to democratize cheminformatics methods for non-experts and simplify their application for the community. MoVIZ includes GUI-guided workflows covering data access, storage, mining, curation, analysis, visualization, modeling, and prediction. This pipeline of workflows is characterized by intuitive prompts, step-by-step instructions, and visual feedback mechanisms. Moreover, MoVIZ incorporates both automated and manual parameter selection options, catering to cheminformatics experts and beginners alike. This flexibility enables users to customize workflows to their needs, ensuring accessibility for all expertise levels.

In this work, we present MoVIZ’s chemical grouping workflow, using supervised and unsupervised machine learning methods. This user-friendly workflow, along with different machine learning approaches and data visualization tools, is made available for download from GitHub (https://github.com/NIEHS/Chemical-grouping-workflow) and KNIME Community Hub (https://hub.knime.com/-/spaces/-/latest/~AnmyNgAW4JMJ_gq4/). It can be deployed on local desktops or network servers. Additionally, the workflow is accessible via the National Institute of Environmental Health Sciences (NIEHS) KNIME Server WebPortal, serving all National Institutes of Health (NIH) users (within the NIH network) as web-application (at https://knime.niehs.nih.gov/knime/webportal/) that also links to other cheminformatics tools and workflows as part of the NIEHS cyber-infrastructure.

In the next sections, we detail the materials and methods used, including data input formats, molecular descriptors, dimensionality reduction, and feature selection techniques. We also describe the implementation of unsupervised clustering and supervised classification methods, supported by hyperparameter tuning for improved performance. The interpretation section showcases the use of SHAP values to provide insights into the importance of molecular descriptors for predicting chemical groupings. Then, we demonstrate the workflow’s capabilities using a large toxicological dataset for eye irritation and corrosion.

## Materials and methods

### Overview of the chemical grouping workflow

There are many motivations for applying chemical groupings, e.g., the chemical groupings can be used to prioritize compounds for inclusion in experimental screening campaigns based on similarity or diversity [46]. In toxicology, chemical groupings are employed to bridge data gaps for compounds with limited information. These compounds are grouped based on their similarity to others with known toxicological properties, suggesting they may exhibit similar toxicological properties. In the same sense, the biological mechanism of action of compounds can be hypothesized [10, 12, 17]. Another application is prior to development of Quantitative Structure–Activity Relationship (QSAR) models, in which the diversity of the compounds in a dataset can be assessed to guarantee that the model will be trained with adequate chemical information and provide reliable predictions for untested compounds [16].

Despite the value of chemical groupings, the literature lacks comprehensive, user-friendly, free and open-source tools for such application. Many available tools are part of paid programs [47], while free options often lack versatility, automation, and guidance [47–50]. This work introduces a comprehensive KNIME workflow designed for chemical grouping. The KNIME analytics platform, being a low-code and no-code platform, facilitates visual programming through the assembly of connected nodes, enhancing understanding and adaptability. All interactive steps and result visualizations are developed using KNIME components, which encapsulate functionality with their own dialog and interactive views. When uploaded to KNIME Server and executed via KNIME WebPortal, the workflow functions as a web application, with each interactive page corresponding to a component in the workflow. Alternatively, the same workflow pages from KNIME components can be accessed locally on the desktop version of the KNIME Analytics platform. KNIME nodes offer configuration options governed by parameters called “flow variables” ensuring dynamic workflow execution. These variables store configurations, parameters, and results, enabling their reuse in future analyses via export to a configuration file.

The main advantages of the chemical grouping workflow described in this work are its versatility, automation, interactive nature, guidance, the number of options it offers at each stage, and ability to support various data formats and a wide range of methods. The workflow provides three different running modes: “New Analysis”, “New Analysis with Prior Configuration”, and “View Past Results”, which satisfy various research requirements.

**Fig. 1:**
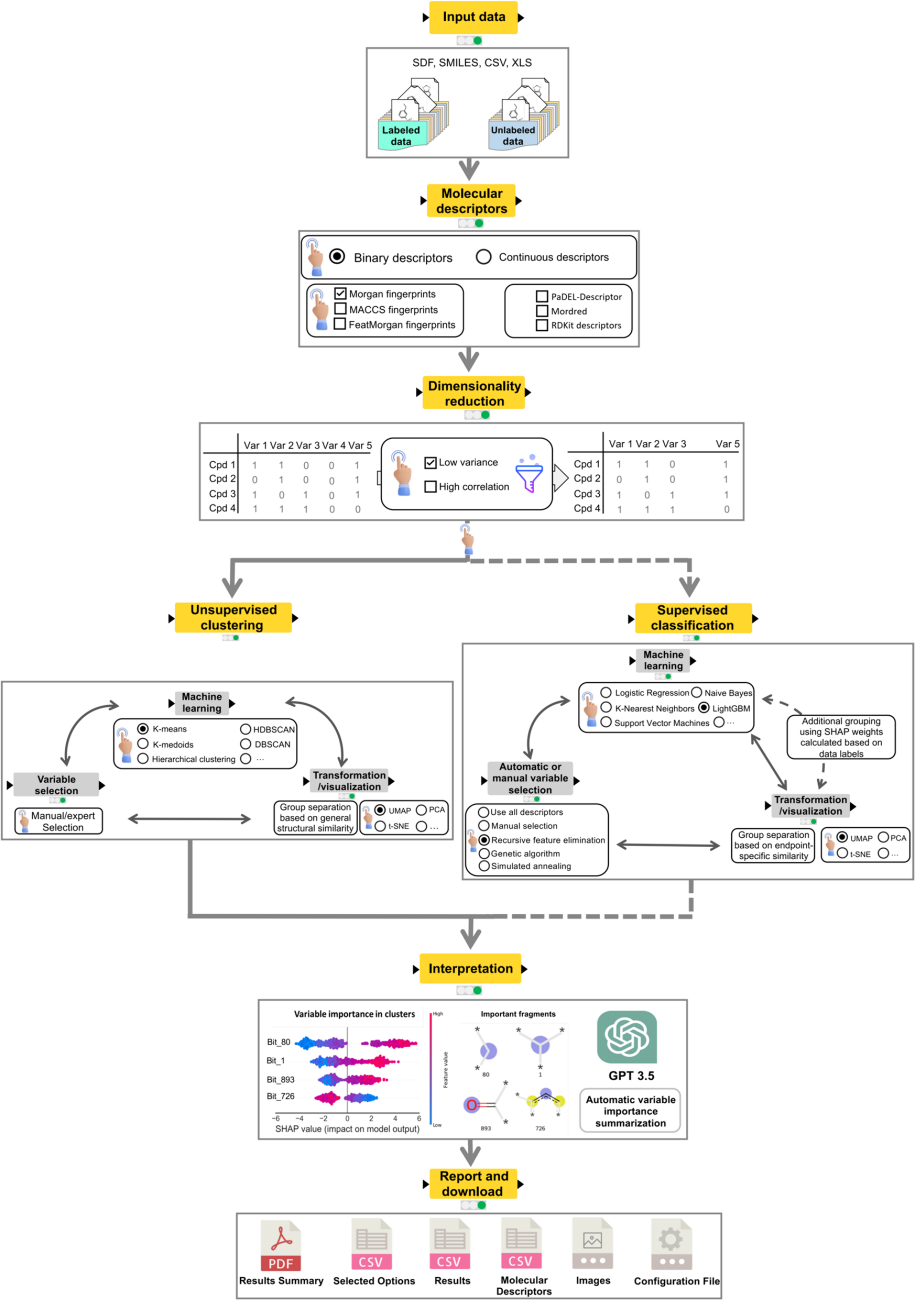
General overview of the chemical grouping process

The default “New Analysis” mode (Fig. 1) allows for chemical data input with or without labels (e.g., biological activity), and supports various file formats like SDF, SMILES, CSV, and Excel (.xls,.xlsx). The workflow standardizes the chemical structures and then calculates molecular descriptors. It offers binary fingerprints or continuous descriptors, and filters low variant and highly correlated descriptors. For unlabeled data running unsupervised clustering, users can manually select features and choose from algorithms like K-means, K-medoids, Hierarchical Clustering, Density-Based Spatial Clustering of Applications with Noise (DBSCAN), and Hierarchical Density-Based Spatial Clustering of Applications with Noise (HDBSCAN), complemented by visualization techniques including Principal Components Analysis (PCA), Uniform Manifold Approximation and Projection (UMAP), and t-distributed Stochastic Neighbor Embedding (t-SNE). For labeled data, supervised classification offers manual and automated feature selection using methods like Genetic Algorithm (GA), Recursive Feature Elimination (RFE), and Simulated Annealing (SA), followed by visualization. A novel option employs SHAP values for finding groups based on endpoint-specific similarity. The workflow further enhances analysis precision by hyperparameter tuning via Bayesian optimization. Results are visualized and interpreted using SHAP plots for feature importance and a large language model (GPT 3.5) for natural language summaries. The final stage produces a comprehensive report with options for detailed downloads, ensuring throughout presentation and utilization of grouping outcomes. Interactive views and guides are available at each step, enhancing user-centric design and accessibility.

After a workflow execution, the system stores all selected options, parameters, and results as “flow variables”, exportable as a configuration file. This file serves as input for the “New Analysis with Prior Configuration” running mode to replicate previous settings for new datasets analyses, ensuring consistency and efficiency. Additionally, the “View Past Results” option leverages this configuration file, enabling users to effortlessly revisit previous outcomes without the re-running the workflow, facilitating in-depth examination of results over time.

### Workflow input

The chemical grouping workflow begins with selecting a running mode: “New Analysis”, “New Analysis with Prior Configuration”, or “View Past Results”, and inputting appropriate files. Each mode accepts different file formats. “New Analysis” initiates a complete new analysis using SDF, SMILES, CSV, or XLS/XLSX files, with CSV and XLS/XLSX requiring a SMILES structures column. For supervised methods, the input file should contain chemical activities encoded in binary, multiclass, or continuous formats. Results, options, and parameters are stored as “flow variables” and exported as a configuration file (.variables) after execution, [51] which can be used as input for other running modes [51].

The “New Analysis with Prior Configuration” is the running mode that uses all the configurations, algorithms, and hyperparameters of a past analysis with a new dataset. This option requires the input of a file with the chemical structures in one of the supported formats (SDF, SMILES, CSV, XLS, or XLSX) and the configuration file (.variables) previously generated. The configuration file is read with the “Read Variables” [52] node. If supervised methods are to be used, it is also required in the file a column with the compounds' activities in the binary, multiclass or in the continuous format. The analysis’ results are also exported as a configuration file.

The “View Past Results” running mode is used to revisit the results generated in a past workflow execution leveraging the interactive visualizations without the need to execute the analysis again. In this option, the only required input is a configuration file previously generated, which will contain all the results.

After inputting of a file containing chemical structures and labels (for supervised analysis), an initial integrity check ensures readability and identifies missing values. Rows with unreadable chemical structures or missing values are removed. A summary table of removed rows is presented and exported as a CSV file. An exploratory analysis follows, displaying the remaining compounds, missing values, and unreadable structures. For labeled data, histograms show continuous data distribution, and class balancing is depicted using bar plots for binary and multiclass data.

### Molecular descriptors

A vital aspect of chemical data analysis and modeling lies in how chemical structures are represented. Although many computer-readable formats for chemical structures exist, the most commonly used format for cheminformatics analysis are molecular descriptors [53, 54]. The molecular descriptors can be calculated from a symbolic representation of a compound or the result of an experimental measurement [55, 56]. In this workflow, we implemented binary and continuous types of molecular descriptors.

Binary molecular descriptors rely on representations in a bit string format where each bit encodes the presence and absence (1 and 0, respectively) of a particular substructure. Here, three different binary molecular descriptors called molecular fingerprints are available through the “RDKit Fingerprint” [57] node: Morgan, FeatMorgan, and Molecular ACCess System (MACCS). The implemented molecular fingerprints are described in the Additional file 1: Table S1.

Continuous molecular descriptors available in the workflow include RDKit descriptors (119 descriptors calculated with the “RDKit Descriptor Calculation” KNIME node [58]), Mordred descriptors (1613 descriptors implemented with the Mordred Python library [59]), and PaDEL-Descriptors (1444 descriptors implemented with the PaDELPy Python library [60–62]). After descriptor calculation, the workflow provides three options for scaling, implemented using the “Normalizer” [63] KNIME node. Min–max scaling linearly transforms descriptor values to a range between 0 and 1. Z-score normalization ensures Gaussian-distribution (i.e., mean is 0 and standard deviation is 1). Normalization by decimal scaling, divides maximum descriptor values (both positive and negative) j-times by 10 until their absolute value is lower or equal to 1. All values are then divided by 10 to the power of j [63].

Another important step of cheminformatics analysis is the data curation and chemical structure standardization. Before the calculation of the molecular descriptors, we implemented the option to apply the QSAR-ready workflow. This performs a standardization procedure on the chemical structures, which includes the removal of salt counterions, stereochemistry, and duplicated entries; standardization of tautomers and nitro groups; correction of valences; and neutralization of structures when possible [64].

### Dimensionality reduction

Implemented dimensionality reduction methods include filtering low variance and highly correlated descriptors, offering manual and automated options. For the low variance filter, descriptors with variance below a set threshold are removed as they lack relevance. In the manual option, the “Low Variance Filter” [65] node removes descriptors based on user defined thresholds. However, selecting an appropriate threshold value can be challenging, particularly for inexperienced users or when numerous descriptors are involved. Hence, an automated threshold search was implemented for labeled data. This involves splitting the dataset into training (80%) and testing (20%) sets, applying variance thresholds ranging from 0 to 0.1, in steps of 0.01 to filter descriptors and generating unique datasets, where machine learning models are trained on the training set and tested for accuracy (for binary and multiclass) or the coefficient of determination [(${R}^{2}$), (for continuous data and regression analysis)] on the test set [66, 67]. The threshold yielding the best model accuracy or ${R}^{2}$ is applied to the original dataset to reduce descriptors. Alternatively, fivefold cross-validation can be employed, dividing the dataset into five distinct splits for comprehensive evaluation of the model's performance. The final performance metric is calculated as the average across these splits.

To filter highly correlated molecular descriptors, the manual option calculates correlation using the “Linear Correlation” [68] node and applies filtering with the “Correlation Filter” [69] node using a user-defined threshold. The automated option follows a similar approach to the low variance filter, splitting the labeled dataset into training (80%) and testing (20%) sets. Correlation thresholds ranging from 0 to 0.1, with increments of 0.01, are applied to both sets using the “corr” method of the pandas Python library [70]. Machine learning models are trained on each dataset, and the threshold yielding the highest accuracy (for binary and multiclass data) or ${R}^{2}$ (for continuous data) is applied to the original dataset. Both options calculate correlation using the Pearson correlation coefficient [71]. Optionally, a fivefold cross-validation process is available, applying each correlation threshold across five dataset splits, with accuracy or ${R}^{2}$ is then calculated as an average across these splits.

### Supervised feature selection

After the removing low variance and/or highly correlated descriptors, feature selection becomes available. It aims to pinpoint a subset closely related to a target physiochemical or biological property while eliminating redundant, noisy, or irrelevant descriptors. Feature selection benefits ML by enhancing model performance and interpretability, reducing overfitting risk, and decreasing training time [72–75]. For unlabeled data, only manual feature selection is available via the “Data Explorer” KNIME node [75]. When data is labeled and supervised classification is selected, feature selection can be manual or automated. Three automated supervised feature selection methods were implemented: Recursive Feature Elimination [76], Genetic Algorithm [77, 78], and Simulated Annealing [79].

In RFE, a machine learning algorithm is trained iteratively starting with all molecular descriptors, then ranks and removes the least important ones until a set number remain [76]. Here, the Recursive Feature Elimination with Cross-Validation method from the scikit-learn [66, 67] Python library was used. At each iteration, the 20% least important molecular descriptors are removed. The final subset is selected based on the highest Area under the ROC Curve (AUC) value for binary and multiclass classification, and the highest ${R}^{2}$ for continuous and regression analysis.

GA is a method that simulates natural evolution and selection for solving complex optimization problems [75, 77, 78], particularly feature selection [73, 80–83]. Employing the sklearn-genetic Python library [84] via the GeneticSelectionCV method, an initial population of molecular descriptors subsets is randomly generated. Each member’s score is determined by training a cross-validated supervised machine learning model, using accuracy or ${R}^{2}$ depending on data type. Tournament selection determines members for to the next generation. Crossover combines selected members to form a new population controlled by “crossover_proba”. Random mutation, controlled by the parameter “mutation_prob”, swaps molecular descriptors. This iterative process continues until the stop criterion, “n_generations”, is met. Users can customize GeneticSelectionCV method parameters, with default values and comprehensive guides provided for ease of use.

SA is also a feature selection method inspired by a natural process [73, 79, 81, 85–88]. This work uses an adaptation from Leung’s implementation [89]. A random selection of 50% of molecular descriptors remaining after dimensionality reduction forms the initial subset. A supervised machine learning model is trained using this subset, evaluating performance via threefold cross-validation using AUC or ${R}^{2}$ scoring. The subset is then iteratively modified by adding, replacing, or removing descriptors, with model performance compared at each step. If a new subset improves performance, it becomes the new current state; otherwise, an acceptance probability is calculated. If the random number is lower than the acceptance probability, the new subset is adopted; otherwise, the original subset is retained. The algorithm’s ‘temperature’ is gradually lowered using a geometric reduction strategy, decreasing by a factor of 0.95 after each iteration. This process continues until reaching a maximum number of 50 iterations or a temperature value below 0.01.

### Chemical grouping

The chemical grouping approach applied in this work is subdivided into two main strategies: unsupervised clustering and supervised classification. The process of grouping chemicals involves the organization of chemical compounds into clusters or classes/categories based on their structural, functional, or property similarities [90]. Unsupervised clustering involves the automatic identification of patterns or similarities among chemicals without any prior knowledge or supervision [90]. It allows us to uncover hidden structures within chemical datasets, revealing inherent relationships between compounds. Supervised classification, on the other hand, utilizes prior knowledge to categorize chemicals [90]. To optimize the performance of both strategies, we employ a rigorous hyperparameter tuning strategy, ensuring that our chemical grouping approach is both robust and effective in capturing the underlying chemical relationships.

#### Hyperparameter tuning

Most machine learning algorithms have tunable hyperparameters that control their behavior and directly affect their performance. Here, we use Optuna [91, 92], a framework-agnostic Python library, to perform automated search for optimal hyperparameters of the machine learning and visualization algorithms for both unsupervised clustering and supervised classification methods. Optuna requires the definition of an objective function containing the entire logic of a standard model’s definition, training, and testing procedure. The objective function returns an evaluation metric. Then, the objective function is optimized to find the best hyperparameters’ combination using the TPESampler (Tree-Structured Parzen Estimator) [93]. The TPESampler is a Bayesian optimization algorithm that run trials iteratively until a user-defined maximum number of trials or time.

The Silhouette coefficient is used as a metric for evaluating the quality of grouping results and selection of the best hyperparameters for the grouping and visualization algorithms. It is implemented using the scikit-learn [66, 67] Python library. It provides a measure of how well data points within a group are separated from points in other groups. It is calculated using the mean intra-group distance (a) and the mean nearest-cluster distance (b) for each sample, as follows:

1 $$SI= \frac{b-a}{\text{max}(a,b)}$$

This coefficient ranges from − 1 to 1, where a value close to 1 indicates that the instance is well-grouped and far from other groups, a value close to 0 indicates that the data points is on or very close to the decision boundary between groups, and a value close to -1 indicates that the data point may have been assigned to the wrong group. Finally, the average Silhouette coefficient across all data points is computed to obtain the overall Silhouette score for the grouping result [94].

The various clustering algorithms and visualization methods have distinct hyperparameters. Therefore, for each algorithm selected by the user, a dedicated page will display the corresponding hyperparameter values for tuning. In the case of Bayesian optimization implemented with Optuna, the user can specify a range of values for each hyperparameter using a slider, and combinations will be tested until reaching a user-defined maximum number of trials. All the hyperparameters available for tuning in the workflow and their respective algorithms are described in Additional file 1: Table S2.

The hyperparameter search process varies based on whether projected clustering is used. Projected clustering involves using a lower-dimensional (typically 2D) subspace generated by visualization methods (described below) as input for the unsupervised clustering and supervised classification algorithms [95–98]. When projected clustering is chosen, both clustering algorithm and the visualization method hyperparameters are tuned simultaneously within a single Optuna run. The 2D projected data output from the visualization method is fed into the clustering algorithm, and the best hyperparameter combination is selected based on the silhouette score. Conversely, without projected clustering, the hyperparameters are tuned first using the entire set of selected molecular descriptors. Subsequently, visualization method hyperparameters are optimized separately. Different combinations of visualization methods hyperparameters are tested, and the resulting 2D projected data from each trial is used as input for the clustering algorithm, while keeping previously tuned hyperparameters fixed. The combination yielding the best silhouette score is selected by default, but users can manually choose a different combination.

#### Unsupervised clustering

Unsupervised clustering in machine learning and data analysis identifies hidden patterns within data by grouping data points based on similarity or density, without using explicit labels (e.g., biological activity). This task relies solely on the data's intrinsic characteristics, assuming that objects within a group share greater similarity than those in separate groups [12–14, 90]. In our grouping workflow, unsupervised clustering employs selected molecular descriptors as input for algorithms such as K-means, K-medoids, Hierarchical clustering, DBSCAN, and HDBSCAN.

K-means [99, 100] is a widely used clustering algorithms that begins by randomly assigning k centroids and then reallocates data points (chemicals) to the closest centroid. It iteratively optimizes centroids by adjusting assignments and selecting new centroids until stability or the maximum iterations are reached [16, 101, 102]. Our workflow implements the K-means algorithm using a combination of the scikit-learn [66, 67] Python library in the hyperparameter optimization and the “k-Means” [103] KNIME node for the final calculation of the grouping.

K-medoids [104] is a modification of the K-means algorithm, where instead of using an artificial data point generated averaging all molecules in the group as the centroid, K-medoids uses the actual middle compound in the group as the center (medoid). Medoids are defined as compounds with the smallest average dissimilarity to all objects within a group [105–107]. This workflow uses the K-medoids implementation of scikit-learn-extra [108] Python library.

Hierarchical clustering algorithm builds groups of molecular compounds using a binary merge tree [109]. Initially, all data points are treated as independent groups (leaves). Then, the closest data points are connected to form pairs, gradually merging into larger groups until reaching the root where all compounds are in a single cluster [21]. The distance between the data points, called “linkage distance”, is computed using single, complete, or average linkage methods. These methods calculate pairwise similarities using Euclidean or Manhattan distance metrics but merge groups differently [110, 111]. The scikit-learn [66, 67] Python library was used for hyperparameter tuning and the “Hierarchical Clustering (DistMatrix)” KNIME node for grouping [111]. The “Hierarchical Cluster Assigner” [112] KNIME node facilitated clustering threshold selection and dendrogram creation.

DBSCAN [113] is an algorithm that groups dense data points, accommodating various shapes and sizes, while identifying outliers. It begins by randomly selecting a data point and measuring the number of nearby points within a limited distance ε (epsilon). If the number of data points inside the ε satisfies a specified minimum (*min_samples*), the initial point becomes a core instance, and all neighboring points are assigned to the same group. This process repeats until all suitable points have been assigned to a group. Data points unassigned to a group are deemed outliers [114, 115].

HDBSCAN [116] extends DBSCAN by introducing groups representation and “robust single linkage”. It calculates point density using a distance metric and constructs a minimum spanning tree for hierarchical clustering. Outliers are handled effectively by labeling them as noise and assigning them to singleton clusters. The algorithm condenses the hierarchy by cutting the tree at a density level determined by the "minimum cluster size" hyperparameter, considering clusters below this size as noise [117, 118]. HDBSCAN is implemented using the hdbscan [119, 120] Python Library.

#### Supervised classification

The unsupervised clustering methods rely on a general concept of chemical similarity. As a result, they might not detect molecular features that most contribute to the target physiochemical or biological property. However, when the data labels are available, e.g., measured toxicity for an endpoint, this information can be used in the grouping process, producing results based on how informative the features are in relation to the target variable [17, 90, 121, 122].

Here, we implemented two methods for supervised classification. The first method starts with the use of one of the supervised feature selection approaches (described above) to find the most relevant molecular descriptors for an endpoint. Then, one of the visualization methods available (described below) are used to project the data into 2D dimensions for visualization. Here, the groups are the data classes (binary or multiclass) or its continuous distributions.

The second supervised classification method uses the SHAP methodology, developed by Scott Lundberg and Su-In Lee [25], based on cooperative game theory and Shapley values [123]. In SHAP, features are treated as "players" contributing to model predictions. It assigns importance values to features by considering all possible combinations, calculating their average marginal contribution to predictions across coalitions [124]. The SHAP Python library computes SHAP values of each molecular descriptor in a trained supervised ML model [25, 125]. These values are then inputted into unsupervised clustering algorithms to group descriptors based on their similarity in influencing the model’s output [97, 121]. SHAP is adapted to data with binary, multiclass, or continuous labels, making it versatile for identifying data groups and subgroups based on labels.

In this work, when data labels are available, the supervised ML methods are used in the automation of the dimensionality reduction and feature selection, and in the supervised classification approach. They are also used in the interpretation of both supervised and unsupervised clustering results (described below). The implemented methods for both classification and regression tasks include Random Forest (RF), Light Gradient Boosting Machine** (**LightGBM), Support Vector Machines (SVM), k-nearest neighbors (KNN). The Naïve Bayesian (NB) and Logistic regression (LR) were implemented only for binary and multiclass classification.

Random Forest is a powerful ensemble learning technique that operates by creating a collection of decision trees, each trained on a random subset of the training data and input features. For classification tasks, it uses majority voting to make predictions, where the class with the most votes from the decision trees is selected. In multiclass classification, this principle is extended to handle multiple classes. For regression tasks, RF calculates the average prediction of the individual decision trees to obtain a continuous numeric output [126–128].

Light Gradient Boosting Machine employs a gradient boosting framework, a popular ensemble method that combines multiple decision trees to create a robust predictive model. It iteratively builds an ensemble of models, each focusing on reducing the errors made by previous models. Final predictions are obtained by summing all models. In multiclass classification tasks, LightGBM extends its boosting capabilities to handle multiple classes effectively. For regression tasks, it provides a continuous numeric output by summing predictions from all models [129, 130].

Support Vector Machines algorithm transforms input data into a higher-dimensional feature space using a kernel function to find a nonlinear decision boundary. It seeks the hyperplane separating transformed data points with the largest margin, traced using nearest data points of each class (support vectors). SVM is effective for multiclass classification tasks and regression problems, where it finds a hyperplane fitting the data points [131, 132].

k-nearest neighbors is a ML algorithm that determines the class membership of the new data by considering its proximity to the existing data points in the feature space. For multiclass classification, KNN extends its nearest neighbor search to find the K nearest neighbors among all classes, and the class with the majority of neighbors is assigned. In regression tasks, KNN calculates the average output of the k-nearest neighbors to provide a continuous numeric prediction [133].

The Naïve Bayesian classifier is a probabilistic classification algorithm based on Bayes’ theorem. It assumes features independence given the class label and calculates class probabilities by multiplying individual feature probabilities. For multiclass classification, NB extends this calculation to estimate class likelihoods, selecting the class with the highest probability. In regression, NB can be adapted to predict continuous numeric values by modifying its probability calculations [134, 135].

Logistic regression is a ML algorithm used for binary and multiclass classification tasks. It is based on the logistic function, also known as the sigmoid function, which maps the input features to a value between 0 and 1, representing the likelihood of the input belonging to the positive class in binary classification. In the case of multiclass classification, LR use its logistic model to handle multiple classes, employing techniques such as one-vs-rest to make predictions across multiple classes [136, 137].

### Data visualization

Data visualization techniques simplify complex multidimensional phenomena by projecting high-dimensional spaces onto lower dimensions. This enables researchers to explore and analyze large datasets effectively, leading to deeper insights [138–140]. To visualize the grouping results into two-dimensional (2D) space, we implemented PCA, UMAP, and t-SNE algorithms. The 2D projected data are plotted using the “Scatter Plot” [141] KNIME node for interactive visualization and the seaborn [142] Python library for static visualization.

PCA reduces the dimensionality of the original dataset while capturing the maximum variance. It creates principal components, which are linear combinations of the original variables. The first component explains the largest variance, with subsequent components explaining the remaining variance while being orthogonal (i.e., no correlation) to each other [143]. We used the “PCA” [144] KNIME node implementation.

UMAP constructs a high-dimensional graph representation of the data and embeds it into a lower-dimensional space using Riemannian geometry. It starts by creating a k-nearest neighbors graph based on a distance metric, then optimizing the embedding process [145]. UMAP was implemented using the umap-learn [145, 146] Python library.

t-SNE calculates pairwise similarities of high-dimensional data, converting them into a probability distribution. It constructs a lower-dimensional space and minimizes the difference between the pairwise similarities of the high-dimensional data and the low-dimensional representation by minimizing the Kullback–Leibler divergence [147]. t-SNE was implemented using the scikit-learn [66, 67] Python Library.

### Interpretation

The interpretation of grouping results is a significant element of our workflow, offering deeper insights and explanation of the grouping rationale. To interpret the grouping results, we train a supervised ML algorithm (classification or multiclass) using molecular descriptors as independent variables and cluster labels as dependent variables. We use the shap [25, 125] Python library to compute SHAP values for each molecular descriptor, indicating their contribution to predicted cluster membership [26–28]. Positive SHAP values denote a positive contribution, while negative values suggest the opposite.

To enhance workflow accessibility and results comprehension, we utilize a large language model (LLM) GPT-3.5 Turbo to distill SHAP value interpretation into concise, coherent summaries. This approach ensures clear and easily digestible explanations for readers across varying levels of expertise.

For Morgan or MACCS fingerprints, we enhance interpretation by generating figures representing molecular substructures linked to individual using the rdkit Python library [148]. We also convert fingerprints bits to corresponding functional groups using the exmol [149, 150] Python library, enriching the input prompt for the LLM. The resulting narrative from the LLM provides a detailed and accessible summary of the grouping results.

For continuous molecular descriptors, two LLM interpretation methods are available. The first method, suited for labeled data, requires a concise description of the endpoint being studied to generate natural language explanations directly linking descriptors’ values to experimental outcomes. The second method, applicable to both labeled and unlabeled data, explanations relating the descriptors’ values to the grouping results without requiring user input on the endpoint.

### Report and download of results

At the end of the workflow a report page is available. In this page, a summary of all results obtained during the workflow execution is shown. Also, options to download the results are provided. These options include:

1. Configuration file (.variables): This file captures parameters and configurations set during the workflow execution, facilitating replication or revisitation of the process. It enables visualization of previous results and ensures consistency for new executions.
2. Workflow parameters and configurations: a CSV file containing execution details such as input file name and extension, data type (labeled or unlabeled), molecular descriptors type (binary or continuous), specific descriptors selected, dimensionality reduction method and the thresholds (low variance, correlation, or all descriptors), grouping analysis type (unsupervised or supervised), feature selection type (manual or automated) with applied algorithm, selected clustering algorithm and visualization algorithms, and hyperparameters after Bayesian optimization or manual selection.
3. Grouping Results: a CSV file that compiles the grouping results (cluster labels, SMILES, and the generated 2D projection of the data for visualization).
4. Grouping results report: a PDF file with the 2D projected data color-coded based on the cluster number, the SHAP summary plot of the grouping interpretation, and the LLM interpretation, when this option is selected.
5. Molecular descriptors: a CSV file with the calculated molecular descriptors that can be used in other applications. If dimensionality reduction and feature selection methods are applied, the CSV file will contain the molecular descriptors remained after those analysis.
6. Figures: all the figures generated during the workflow execution including the static scatter plot and the SHAP summary plots in high resolution (SVG format).
7. Data failed during the input: a CSV file that includes data which was either unreadable or had missing values during the input process.

### Documentation and guides

The utility of a scientific application is significantly enhanced by comprehensive documentation, a critical aspect that extends beyond mere technical robustness. Such documentation ensures that the application is not only reproducible but also user-friendly, making complex processes approachable and comprehensible. This clarity and ease of use are vital in breaking down barriers to entry, allowing researchers from various backgrounds to use the tool effectively. Furthermore, comprehensive documentation and guides are a cornerstone in the democratization of scientific tools and methods to ensure that these resources are not only used by experts in the field but are accessible to a broader community. This inclusive approach promotes collaborative research, encourages a diversity of perspectives, and accelerates scientific discoveries [35, 151, 152].

In this study, documentation was embedded directly within the KNIME workflow to facilitate this comprehension and reproducibility. Each node, metanode, component, or Python script within the workflow was annotated with detailed comments. These comments encompassed a description of the function, and any specific methods or algorithms being employed within. In cases where sequences of nodes worked together to accomplish a specific task, metanodes were employed, and comments were added to these to describe the collective functionality.

All the steps of the workflow execution possess interactive pages designed to enhance user guidance and accessibility. At each step of the workflow, informative text boxes serve as guides to provide users with clear and concise instructions, leading them through the various stages of the chemical grouping process with step-by-step guidance.

### Computational tools

The workflow was developed using the open-source software KNIME version 4.6.4 (freely available at https://www.knime.com/download). The KNIME free extensions used were: “KNIME Base Chemistry Types & Nodes” [153], “Indigo KNIME Integration” [154], “RDKit Nodes Feature” [155], “KNIME Python Integration” [156], “KNIME JavaScript Views (Labs)” [157], “Vernalis KNIME Nodes” [158], and “KNIME HTML/PDF Writer” [159]. Some parts of the workflow were implemented in “Python Script (legacy)” nodes and Python 3.10.13 using the following libraries: pandas (v. 1.5.2) [70], numpy (v. 1.25.3) [160], mordred (v. 1.2.0) [59, 161], PaDELPy (v. 0.1.14) [61], scikit-learn (v. 1.0.2) [66, 67], LightGBM [129, 162], sklearn-genetic (v. 0.6.0) [84], shap (v. 0.41.0) [25, 125], hdbscan (v. 0.8.28) [119, 120], optuna (v. 3.0.2) [91, 92], rdkit (v. 2022.3.5) [148], umap-learn (v. 0.5.3) [145, 146], cairosvg (v. 2.7.0) [163], scikit-learn-extra (v. 0.2.0) [108], seaborn (v. 0.11.2) [142], ipython (v. 7.34.0) [164], pillow (v. 9.4.0) [165], openai (v. 1.10.0) [166], exmol (v. 3.0.3) [149, 150], and matplotlib (v. 3.6.3) [167].

The workflow is deployed on the NIEHS KNIME Server (v. 4.15.3, running on CentOS Linux v. 3.10.0) and made available via the KNIME WebPortal to be executed as a web application in a guided step-by-step way, without the need to install the KNIME analytics platform. Currently, the NIEHS KNIME server is only available within the NIH network at https://knime.niehs.nih.gov/knime/webportal/. But the same workflow can be downloaded from GitHub (https://github.com/NIEHS/Chemical-grouping-workflow) or KNIME Hub (https://hub.knime.com/-/spaces/-/latest/~AnmyNgAW4JMJ_gq4/) and executed locally or deployed to any other KNIME Server.

### Case study

To demonstrate the functionalities and outputs of the chemical grouping workflow, we performed two separate analyses: a supervised classification and an unsupervised clustering. The toxicological eye irritation and corrosion dataset retrieved from the work of Borba et al. [168] was used for both analyses. This dataset was downloaded from the supplementary material of the original publication, containing a total of 2273 chemicals, comprising 1140 irritants and 1133 non-irritants.

## Results and discussion

### Workflow overview and execution

The chemical grouping workflow described above comprises nine major steps: (1) data input; (2) molecular descriptors calculation; (3) dimensionality reduction; (4) feature selection; (5) hyperparameter tuning; (6) chemical grouping; (7) results visualization; (8) interpretation of the results; and (9) reporting.

**Fig. 2:**
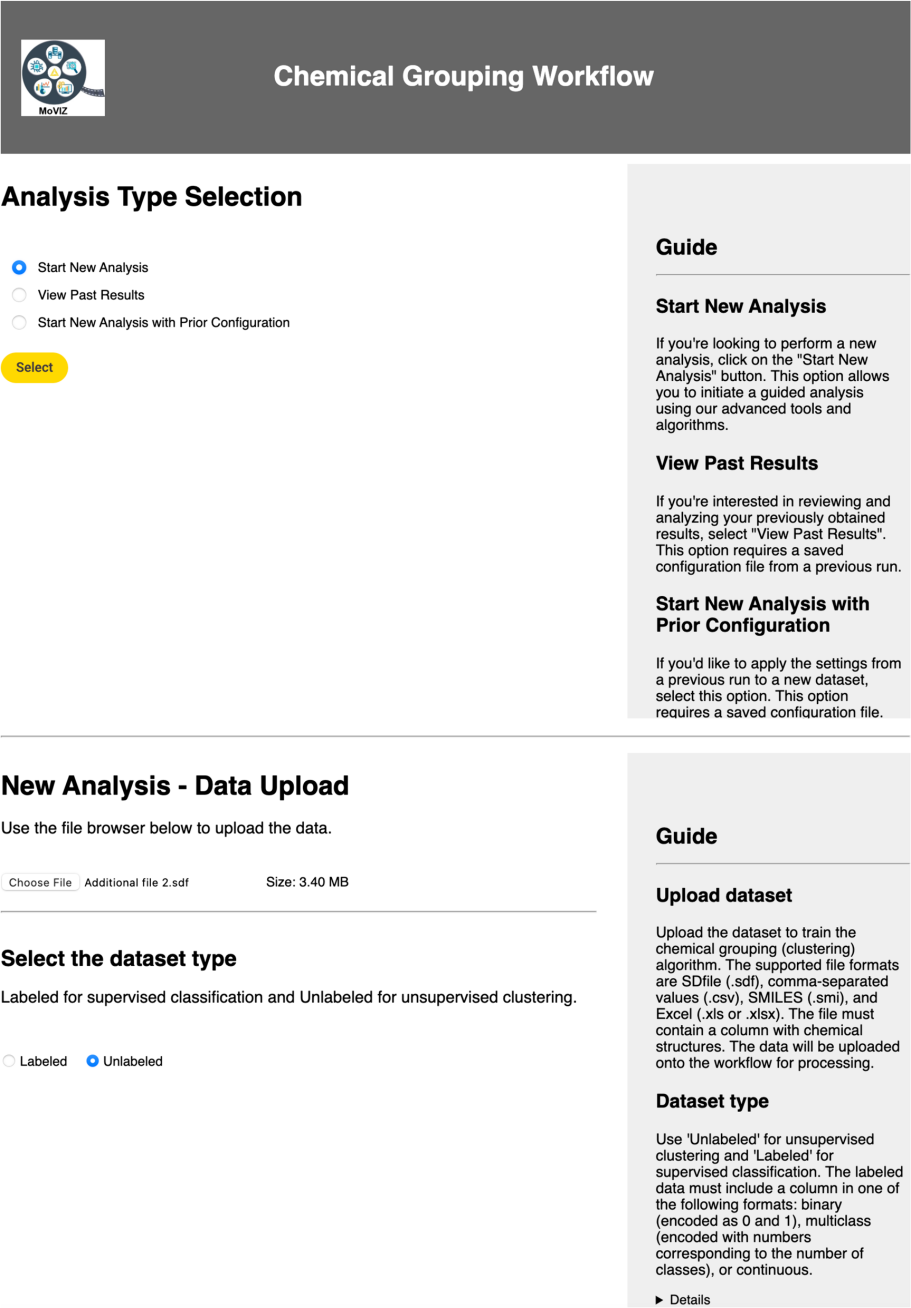
Input and initial configuration page of the chemical grouping workflow, with detailed user guides

The in-workflow documentation ensures intuitive understanding of the logic and methodology being employed at each stage, without the need for external documentation or guesswork, and supports future modifications and extensions to the workflow. Each interactive page view of the workflow presents a text box on the right side for guidance (Fig. 2). It provides instructions for executing the workflow (including any required user input or potential parameter adjustments), and guidance for interpreting the output results. Where necessary, the interactive view documentation also provides links to more detailed external resources, such as academic papers and technical documentation for the methods. This dual-pronged approach to documentation, i.e., combining in-workflow comments with a step-by-step guide in the interactive page view, was designed to make the chemical grouping workflow as understandable and user-friendly as possible, while still providing the depth of information required for full reproducibility and potential future development.

### Input and exploratory analysis

In our case study, the “New Analysis” mode was selected (Fig. 2). The input file (Additional file 2) was in the SDF format, containing the chemical structures of the eye irritation and corrosion dataset. We first performed an unsupervised clustering analysis with this dataset, using the “Unlabeled" option under the “Select dataset type” configuration field.

We also used the same dataset for a supervised classification analysis, on a second workflow run using an SDF file containing the chemical structures and the labels for the eye irritation and corrosion dataset, encoded as zero for nonirritant chemicals and one for irritants. To unlock the supervised methods in the workflow, the “Labeled” option under the “Select the dataset type” field was chosen (Fig. 2). Then, we selected the SDF column “Outcome” containing the labels and the data type “Binary” in the “select column” page (Additional file 1: Figure S1). Next, the exploratory data analysis revealed that four chemicals could not be read by the workflow, resulting in a final dataset of 2269 chemicals, with 1137 classified as class zero and 1132 as class one (Additional file 1: Figure S2).

### Chemical grouping

#### Unsupervised clustering

**Fig. 3:**
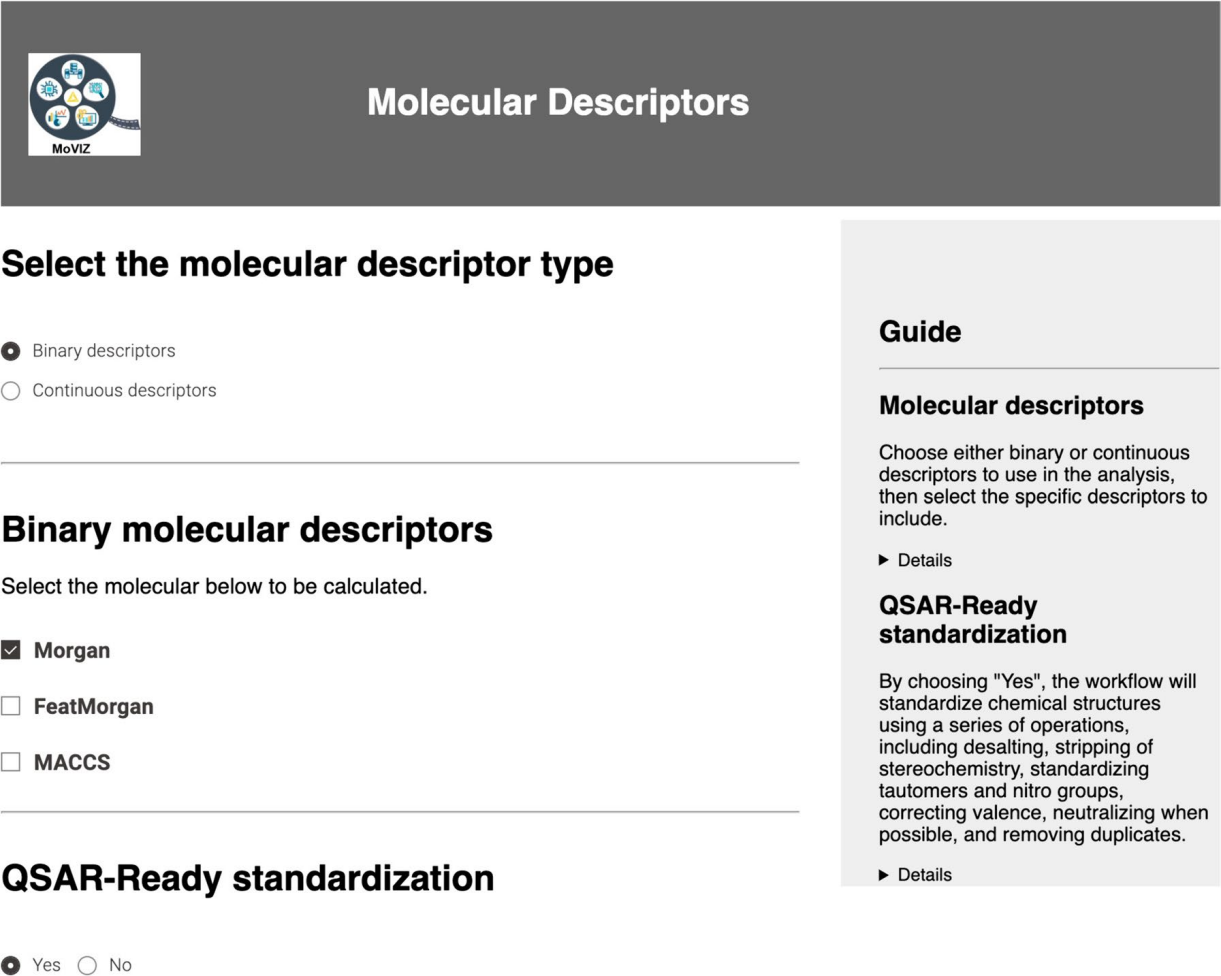
Page for the molecular descriptor selection and chemical structure standardization

In the unsupervised clustering analysis of our case study using the eye irritation and corrosion dataset, the binary Morgan fingerprints was selected (Fig. 3). The Morgan parameters set were the “radius” = 3 and “number of bits = 2048 (Additional file 1: Figure S3). The "QSAR-ready standardization" option was set to "Yes" by default to apply the chemical structure standardization steps of the QSAR-ready workflow [64].

**Fig. 4:**
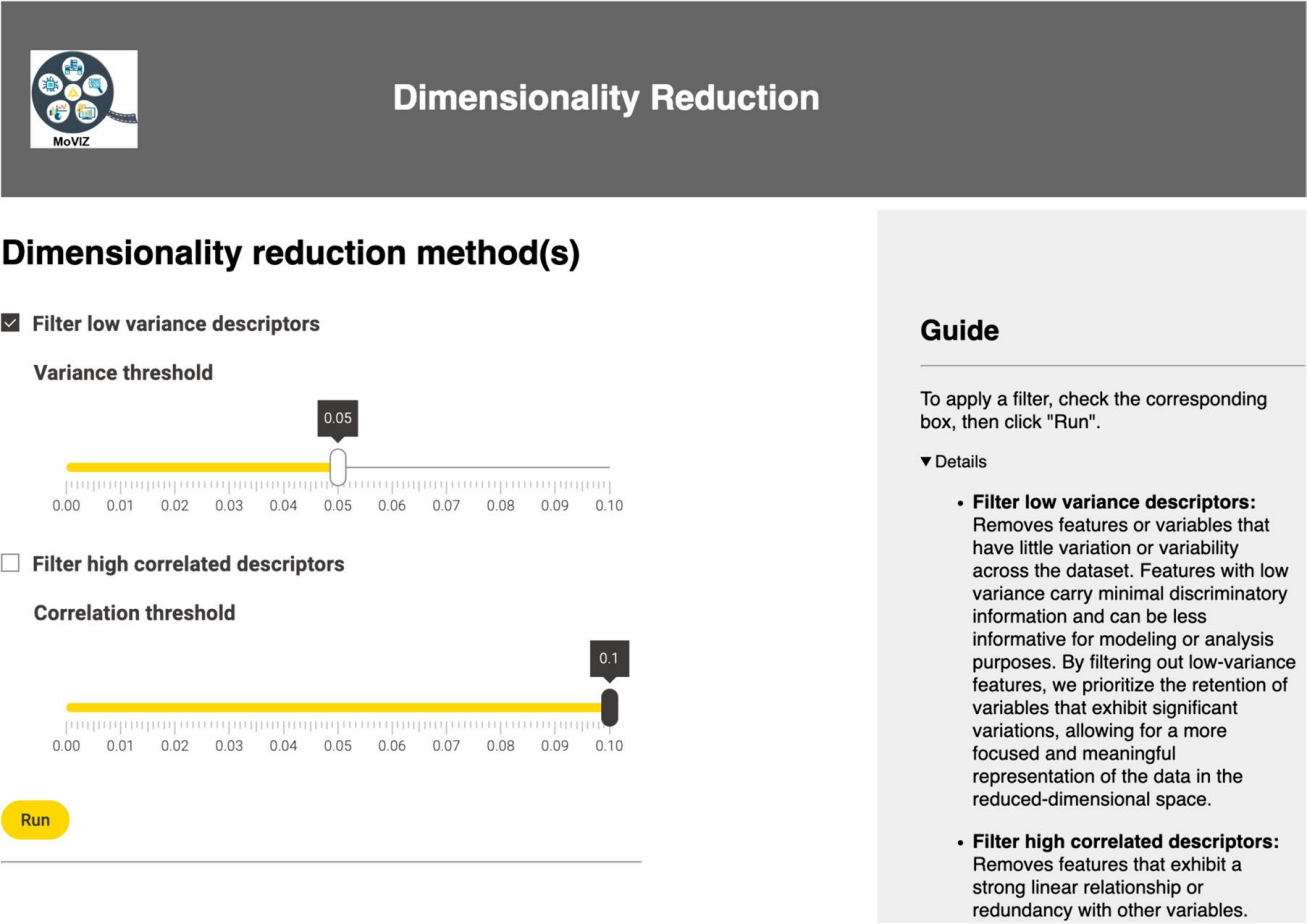
Configuration page of the manual option for the dimensionality reduction

Upon the calculation of the molecular descriptors, the next step is the dimensionality reduction. In the unsupervised clustering analysis, we applied the low variance filter and manually selected the threshold using a slider (Fig. 4). The selected threshold was 0.05 and 92 bits from 2048 remained after the filtering. For the feature selection step, when the input data are unlabeled or the user selects the unsupervised clustering method, only a manual option is shown (Additional file 1: Figure S4). The user can manually select in a table the binary fingerprints or molecular descriptors that will be removed from the dataset. Also, the univariate statistical measures Mean, Variance, Skewness, and Kurtosis are calculated and displayed in the table for each fingerprint or molecular descriptor to guide the user in the selection. In our unsupervised clustering case study, no additional feature selection was performed after the dimensionality reduction and the 92 bits were used in the subsequent analysis.

After the feature selection, the next page is the chemical grouping configuration. In this page, the selection of the clustering algorithm (K-means, K-medoids, Hierarchical clustering, DBSCAN, and HDBSCAN), the visualization method (UMAP, PCA, and t-SNE) and the use of projected clustering are available (Additional file 1: Figure S5). The default configurations are K-means, UMAP and “Yes” for the use of projected clustering. The projected clustering methods act as a preprocessing step for the clustering algorithm to overcome the “curse of dimensionality” problem, i.e., when the algorithm have a poor performance due to the high-dimensional space of the feature set. Thus, the grouping performance is increased [96, 97, 169, 170]. For the unsupervised clustering analysis of our case study, the default configurations were used.

**Fig. 5:**
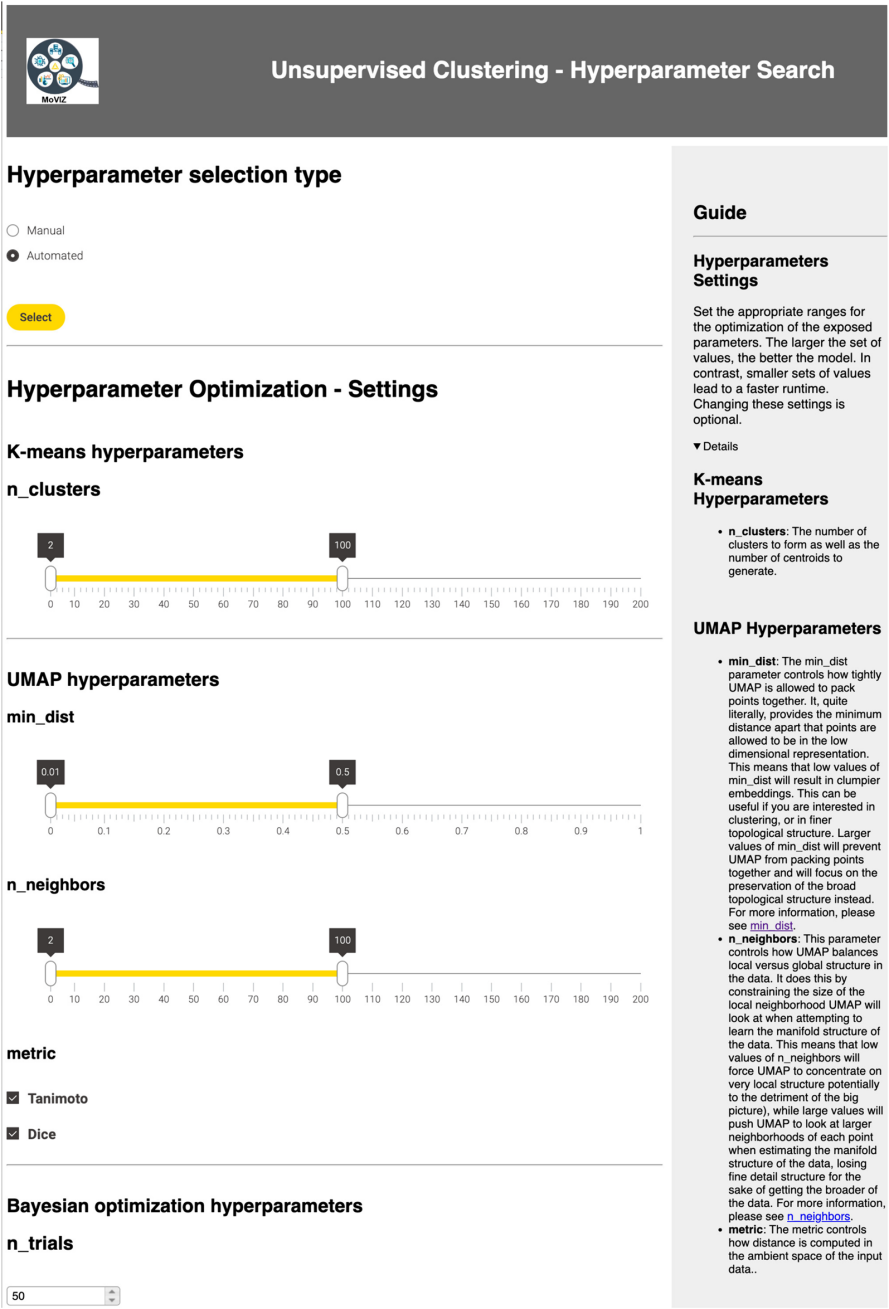
Configuration page displaying the hyperparameters’ options for the K-means clustering algorithm and the UMAP visualization method of the unsupervised clustering analysis. The page features sliders to specify ranges for each hyperparameter when using Bayesian optimization with Optuna. The displayed hyperparameters’ ranges were used in the case study

After the chemical grouping configuration page, the next step is the hyperparameter tuning. The configuration page for the K-means clustering algorithm's hyperparameters, along with the selected range values for tuning, is displayed in Fig. 5. The combination of hyperparameters that resulted in the highest Silhouette score (0.63) was selected: *n_clusters* = 3 for K-means, and *min_dist* = 0.02 and *n_neighbors* = 17 for UMAP (Additional file 1: Figure S6).

**Fig. 6:**
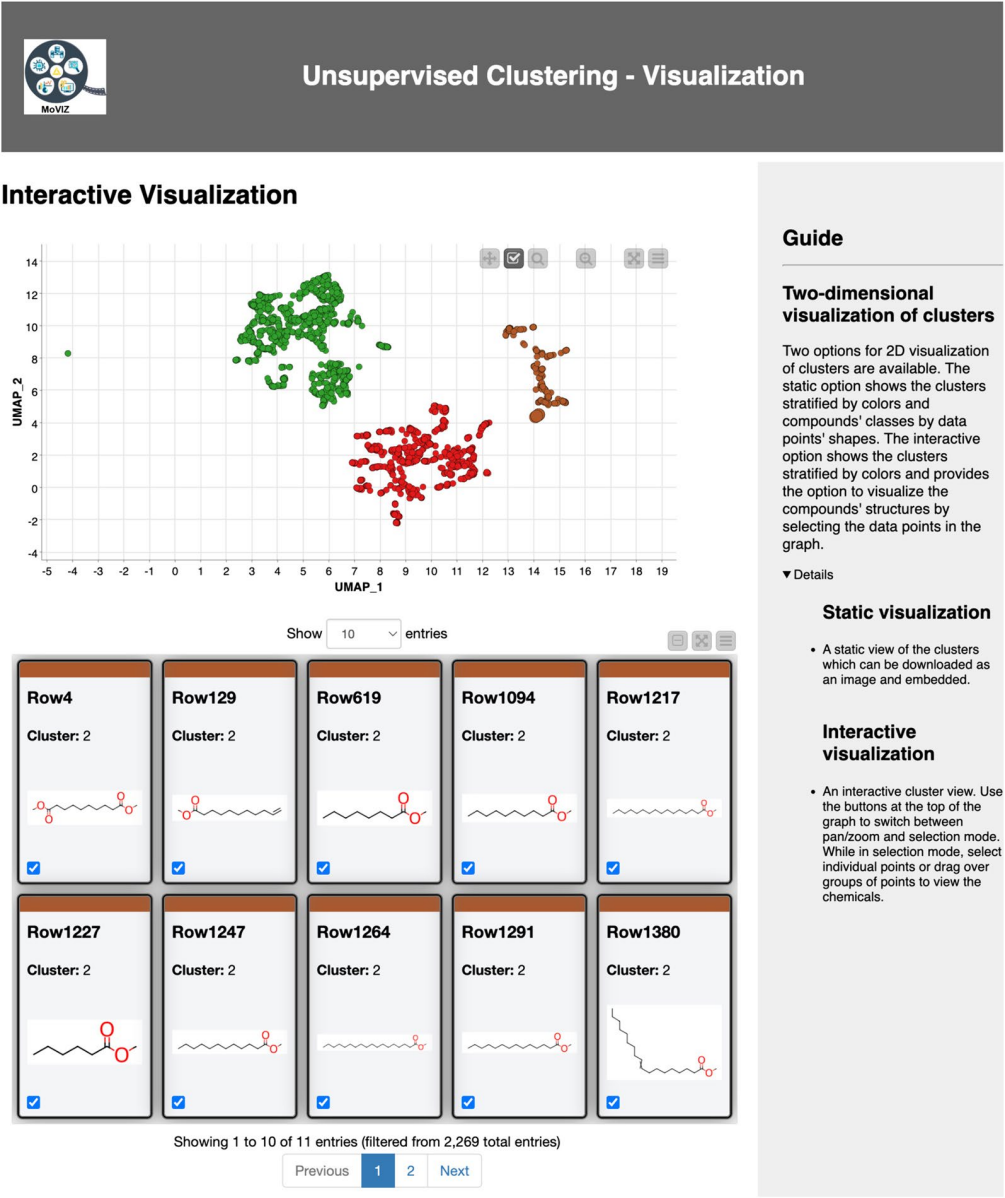
Unsupervised clustering results. The interactive scatter plot of the 2D projection is shown. Each cluster is identified by a different color in the chart. The user can interactively select data points in the scatter plot and visualize the respective chemical structures

The visualization of the grouping results is performed using an interactive scatter plot of the 2D projected data where the data points (chemicals) are color-coded based on the group (cluster) number (Fig. 6). In the interactive scatter plot, the user can select data points and visualize their chemical structures. As we can observe in the highlighted chemicals within cluster number 2 in Fig. 6, unsupervised clustering algorithms group molecules based on structural similarities since they utilize unlabeled data. The applications of unsupervised analysis are diverse; for example, they can be used to analyze the similarity and chemical space of a dataset or database, to select and prioritize chemicals for experimental testing, or to generate cluster-based splits for training predictive machine learning models.

It is important to note that the selection of a chemical grouping approach depends on the objectives and context of the study, which, consequently, will influence the choice of the type of analysis (unsupervised or supervised), the selection of molecular descriptors, grouping algorithm, and hyperparameters. Therefore, there is no single approach or method that can definitively determine the "optimal" groups universally. Consequently, researchers frequently analyze outcomes from various approaches [90, 171]. In this sense, the in-workflow guides, the options for automated variable selection and hyperparameter searches, the interactive visualization and interpretation of the results, and the different options for results downloads and exportation aim to help the user in the grouping process.

**Fig. 7:**
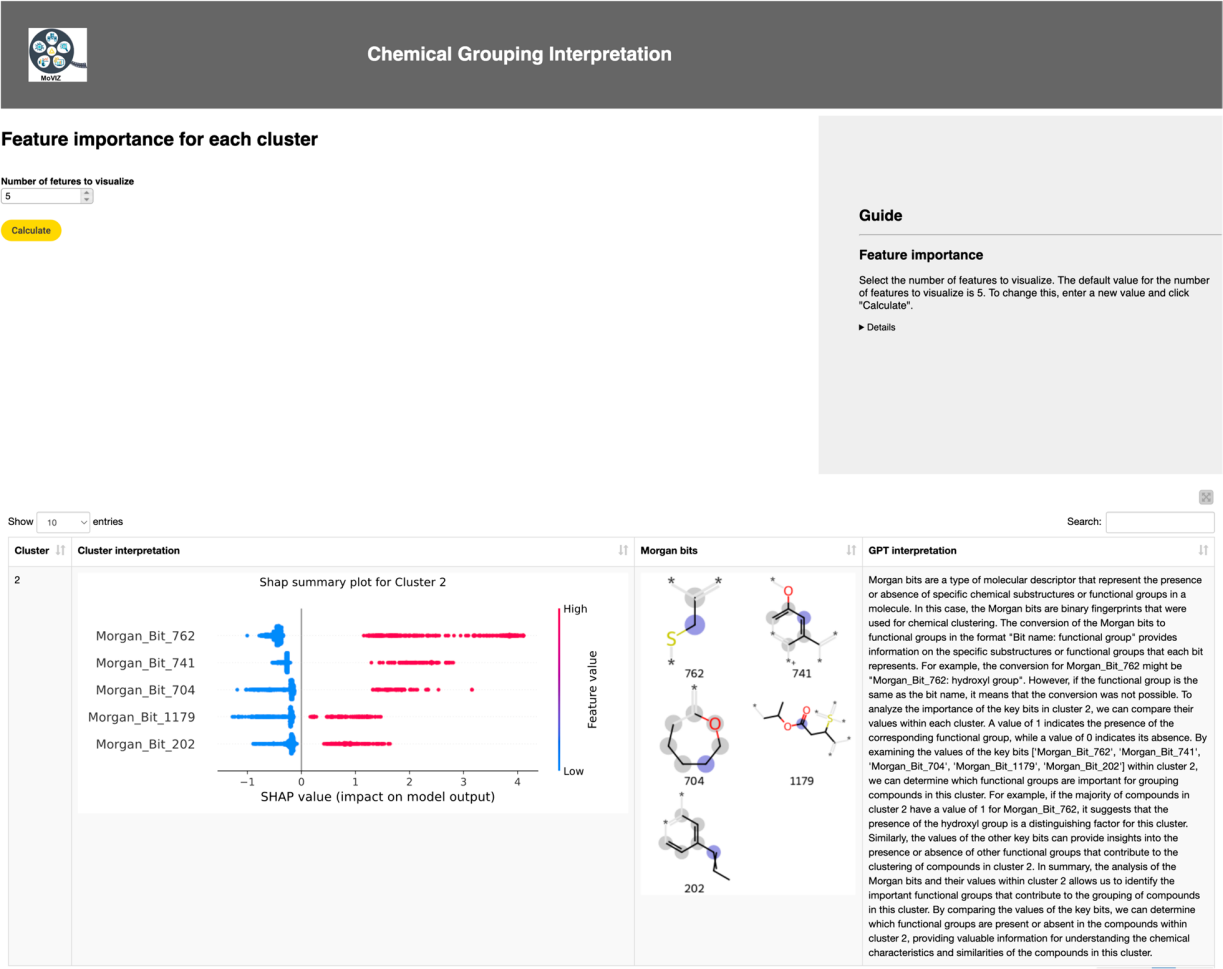
Interpretation of the cluster number 2 identified using the unsupervised clustering method

Following the visualization of the grouping results, the next step is the interpretation using SHAP summary plots and the natural language explanation generated by the LLM (GPT 3.5 Turbo). The use of GPT-3.5 Turbo is optional; to utilize it, the user must input an OpenAI API key, as described in the in-workflow guide (Additional file 1: Figure S7). In the SHAP summary plot, the most important molecular descriptors are ranked from top to bottom in the y-axis. On the x-axis, for each molecular descriptor, every compound on the respective group appears as a data point horizontally distributed according to their SHAP values. These plots additionally display the influence of the bits on the model prediction by color (red for presence of a substructure and blue for the absence). In the interpretation of the unsupervised clustering results depicted in Fig. 7, we identify the most important bits that group chemicals in cluster number 2 (the interpretation of the results for all clusters is shown in Additional file 1: Figure S8). Taking bit 1179 as an example, which possesses an ester group, we observe that all the highlighted chemicals in Fig. 6 present this functional group. This information is instrumental in interpreting the chemical diversity of the dataset and identifying if the dataset is biased to specific chemical scaffolds. This analysis can be further augmented by incorporating other available information, such as chemical labels, to assist in inferring structure–activity relationships.

#### Supervised classification

**Fig. 8:**
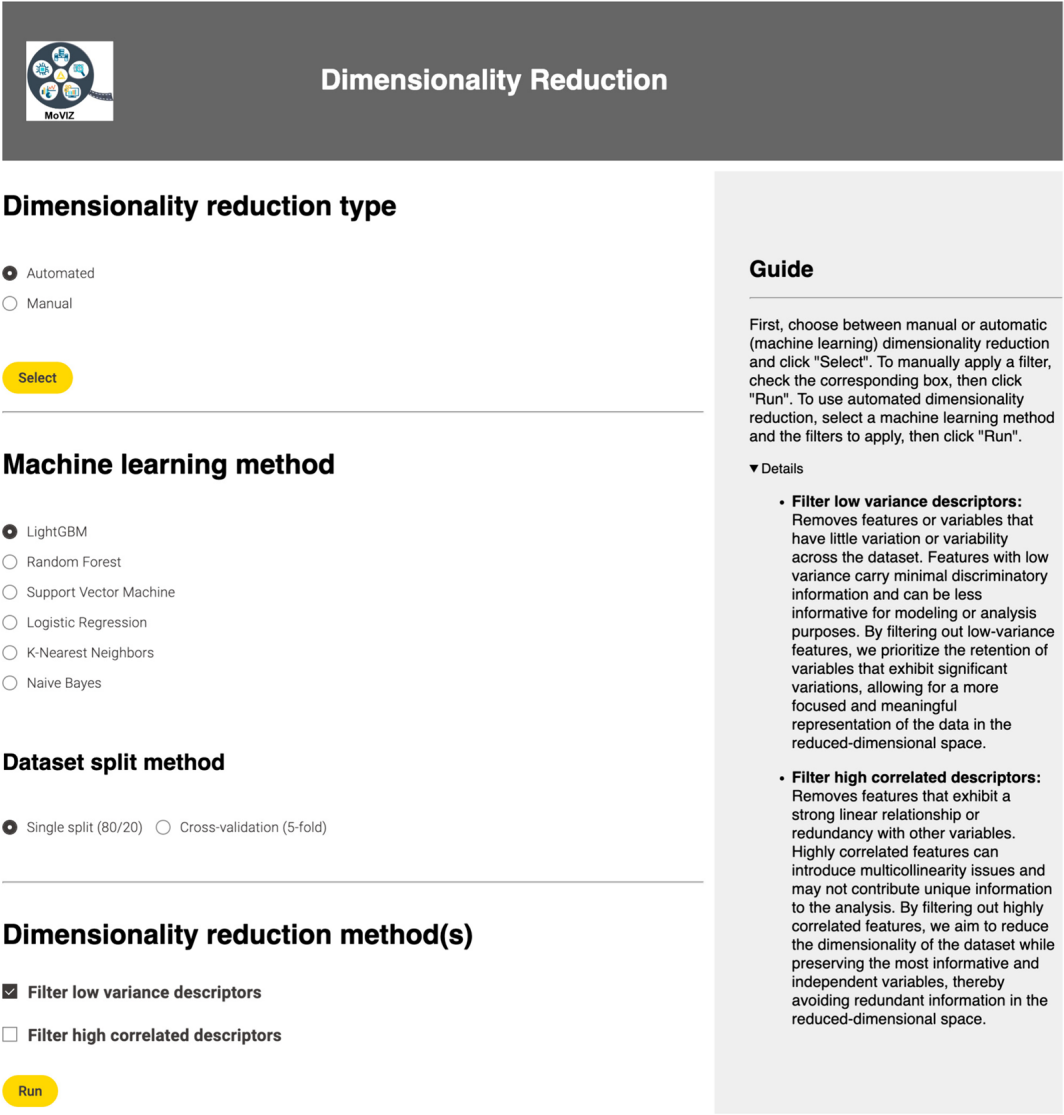
Configuration page of the automated option for the dimensionality reduction step

In the supervised classification of our case study using the eye irritation and corrosion dataset (1137 non-irritants and 1132 irritants), the continuous molecular descriptors option and the Mordred descriptors was selected (Additional file 1: Figure S9). Subsequently, we filtered out the low variance descriptors using the automated option (Fig. 8). The LightGBM algorithm was used to find best variance threshold (0.03). The selection of a supervised ML algorithm appears only in the first step requiring this method. All later steps use this same algorithm. After the application of the filter, 747 descriptors from the initial number of 1051 remained.

**Fig. 9:**
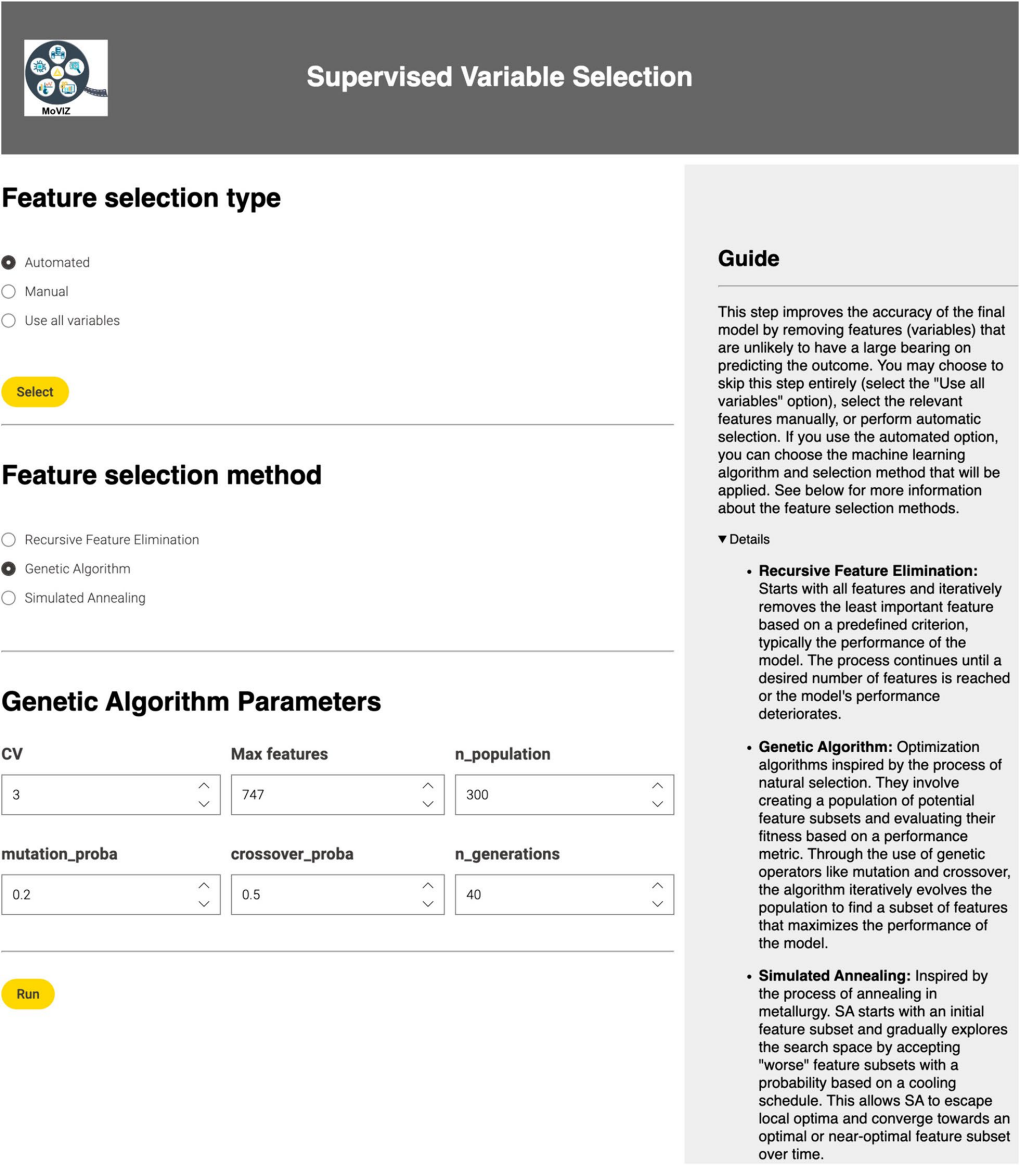
Configuration page for automated feature selection

Following the removal of low variance descriptors, the automated feature selection using the Genetic Algorithm method was employed (Fig. 9). This method automatically selects the best subset of molecular descriptors related to the endpoint for chemical grouping. From the 747 descriptors remained after the automated dimensionality reduction, 74 descriptors were selected and used as the molecular descriptors for chemical grouping.

In addition to the automated feature selection, for our supervised classification we applied the SHAP method to calculate the weight of each molecular descriptor (SHAP values) based on the labels of irritant or nonirritant. Then, we used the projected clustering method with UMAP as the visualization algorithm to project the SHAP values in a 2D space, which was used as input for the unsupervised clustering algorithm. Here, we selected K-medoids to demonstrate the capabilities of the chemical grouping workflow. Additional file 1: Figure S10 displays the configuration page for the K-medoids clustering algorithm, including the hyperparameters and their selected range values for tuning. By default, a broad range is chosen for the hyperparameters to accommodate various purposes, but users can modify these values and the number of combinations to be tested by the Bayesian search. We limited the search of *n_clusters* from 2 to 25 for K-medoids, and for UMAP the *min_dist* of 0.01–0.25 and *n_neighbors* of 2–50. The number of trials for the Bayesian search was 50. After the hyperparameter tuning, the following combination of hyperparameters was selected: *n_clusters* = 9 for K-medoids, and *min_dist* = 0.02 and *n_neighbors* = 32 for UMAP, resulting in Silhouette score of 0.42 (Additional file 1: Figure S11).

**Fig. 10:**
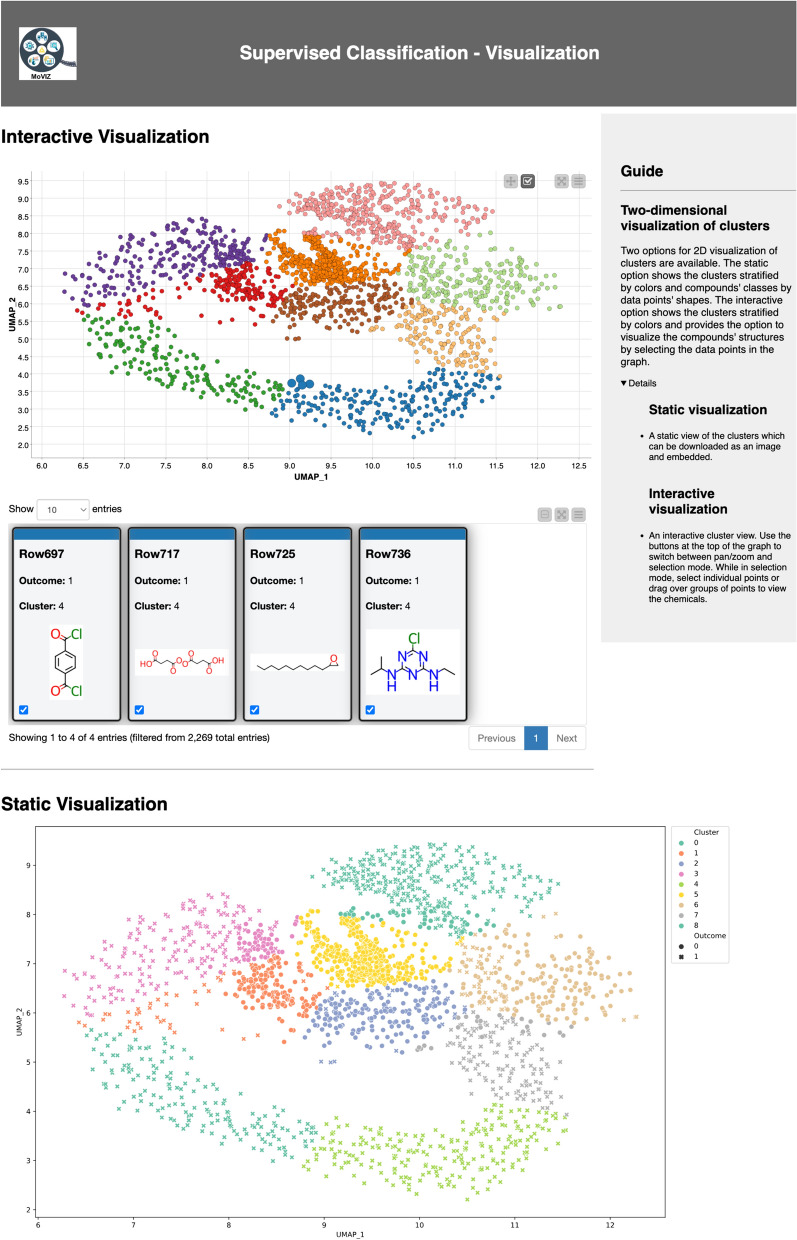
Supervised classification results. The interactive and static scatter plots of the 2D projected are shown. Each cluster is identified by a different color in the charts. The user can interactively select data points in the interactive scatter plot and visualize the respective chemical structures. In the static plot, the nonirritant data points are dot-shaped and irritants are x-shaped

The grouping results are visualized using scatter plots of the 2D projected data with two options: interactive and static (Fig. 10). In both options, the data points (chemicals) are color-coded based on the group (cluster) number. In the interactive option, the user can select data points in the scatter plot and visualize their chemical structures and outcomes (1 = irritant and 0 = nonirritant), and also perform zoom in and out in the scatter plot. In the static option, in addition to color-coding of the groups, the data points are also shape-coded based on the outcomes (here, eye irritation and corrosion). As we can see in the Fig. 10, using the data labels and supervised algorithms, the supervised classification method implemented was able to group chemicals based on endpoint-specific similarity. Clusters 0, 4, 7, and 8 have a high proportion of irritants. Conversely, clusters 1, 2, 5, and 6 have a higher proportion of non-irritants. Cluster 3 shows a mix, with a higher prevalence of irritants than non-irritants.

**Fig. 11:**
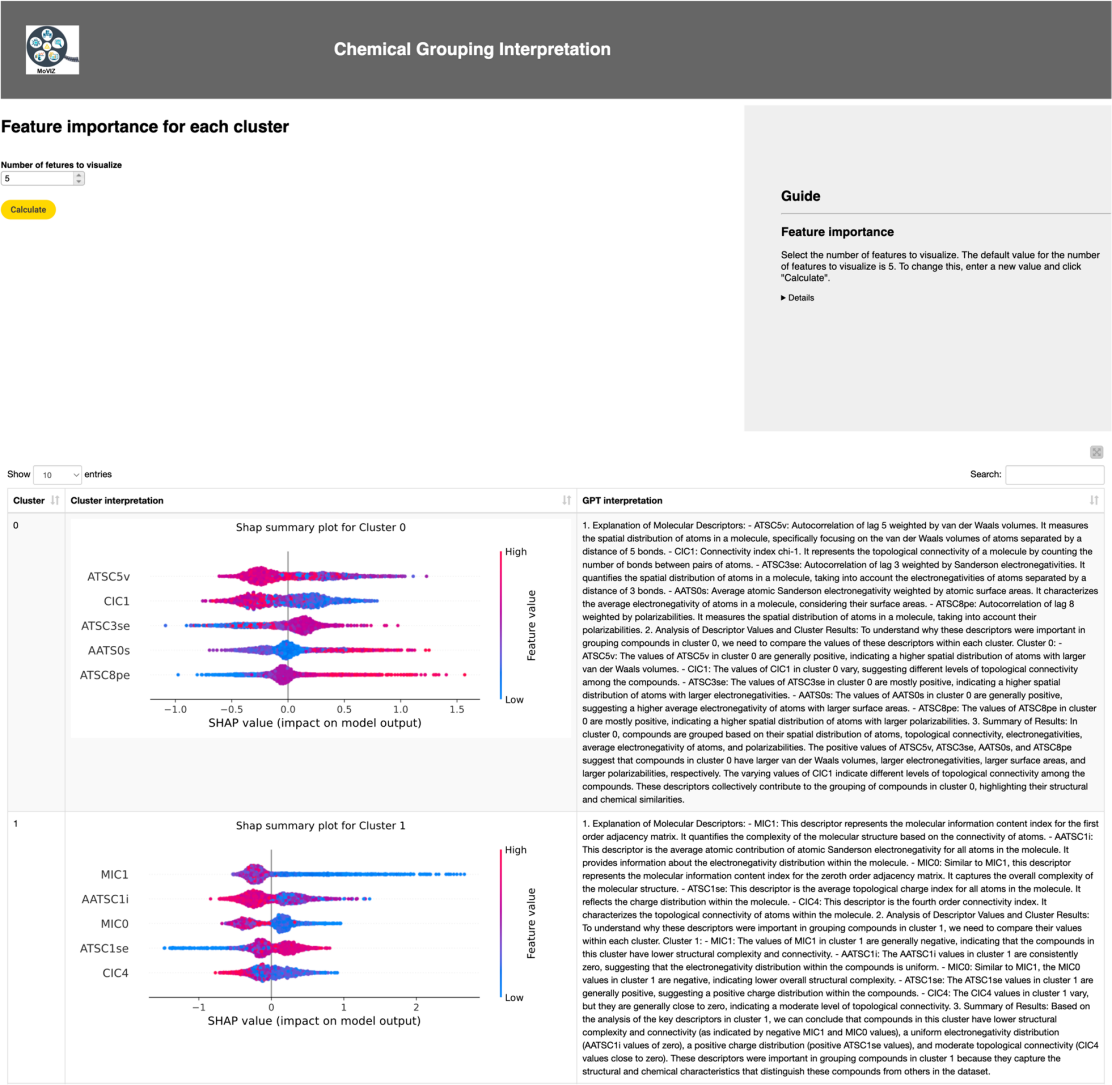
Interpretation of the clusters number 0 and 1 identified using the supervised classification method

In the interpretation of the supervised classification results, we show only the interpretation of the clusters number 0 and 1 (Fig. 11). The complete results are shown in the Additional file 1: Figure S12. SHAP values were utilized to identify the top 5 most important molecular descriptors for each group identified. The SHAP summary plot also display the influence of the molecular descriptors' values on the model prediction through the use of color. For the cluster number 0, the most important descriptors to group the chemicals were ATSC5v (centered Moreau-Broto autocorrelation of lag 5 weighted by van der Waals (vdw) volume), CIC1 (1-ordered complementary information content), ATSC3se (centered Moreau-Broto autocorrelation of lag 3 weighted by Sanderson EN), AATS0s (averaged Moreau-Broto autocorrelation of lag 0 weighted by intrinsic state), and ATSC8pe (centered Moreau-Broto autocorrelation of lag 8 weighted by Pauling EN) (see Fig. 11). Since the cluster number 0 is predominantly formed by irritants or corrosive chemicals, this suggests that the molecular descriptors identified as important in this cluster may be associated with eye irritant/corrosive properties. On the other hand, the cluster number 1 is predominantly formed by non-irritants/noncorrosive chemicals (see Fig. 10), indicating that the distinguishing molecular descriptors in this cluster [MIC1 (1-ordered modified information content), AATSC1i (averaged and centered Moreau-Broto autocorrelation of lag 1 weighted by ionization potential), MIC0 (0-ordered modified information content), ATSC1se (centered Moreau-Broto autocorrelation of lag 1 weighted by sanderson EN), CIC4 (4-ordered complementary information content)] might be linked to properties that reduce the likelihood of being an irritant or corrosive (see Fig. 11). This information can be valuable for researchers in several applications regarding this endpoint, e.g., in the development of new chemicals with ocular exposure potential.

One drawback of using continuous molecular descriptors is that the computational libraries used to compute them often only output names and values. These names are often difficult to understand, requiring extensive literature research to identify the molecular properties each descriptor calculates. Additionally, interpreting the SHAP summary plot may not be straightforward for inexperienced users. To address these issues, the large language model GPT-3.5 Turbo was utilized to automatically generate explanations for the most important descriptors in each cluster and provide a natural language summary of the interpretation results. As seen in Fig. 11, the LLM starts by explaining the key molecular descriptors. It then correlates these descriptors' values with the clustering results. Finally, it provides a summary of these findings. For example, the summary for cluster number 0 states: "*In cluster 0, compounds are grouped based on their spatial distribution of atoms, topological connectivity, electronegativities, average electronegativity of atoms, and polarizabilities. The positive values of ATSC5v, ATSC3se, AATS0s, and ATSC8pe indicate compounds in cluster 0 have larger van der Waals volumes, electronegativities, surface areas, and polarizabilities, respectively"*. These descriptors collectively contribute to grouping the compounds in cluster 0, highlighting their structural and chemical similarities. This grouping is significant, considering that cluster 0 mainly consists of irritants/corrosives, and these properties may correlate with the endpoint, and they are consistent with published data [172–174]. The LLM-generated explanations for all clusters are available in Additional file 1: Table S3. Thus, the LLM's automatic explanation generation significantly contributes to the democratization and facilitation of chemical grouping analysis.

### Report and results download

The final step of the workflow is a summary showing all results obtained during the process, with options to download the results obtained (Additional file 1: Figure S13). In the unsupervised clustering analysis of the eye irritation and corrosion dataset, the binary Morgan fingerprints were used, and bits were filtered using a manually set variance threshold of 0.05. We used the K-means clustering algorithm and UMAP for visualization. The analysis identified 3 clusters with a Silhouette score of 0.63. This information, along with other relevant parameters, is summarized in the ‘Selected Options’ field and is available for download in CSV format. Additionally, the SMILES of the compounds in the dataset, the UMAP coordinates, the cluster labels, and the molecular descriptors are available for download in CSV format. The complete page for report and results download of the unsupervised clustering analysis is shown in the Additional file 1: Figure S14.

In the supervised classification analysis of the eye irritation and corrosion dataset, we used the Mordred continuous descriptors, and the dimensionality reduction (filter of low variance and high correlated descriptors) was performed using the automated option. Subsequently, the best subset of descriptors for the studied endpoint was selected using GA, and the SHAP method was applied to weigh them based on the data labels. All supervised methods used the LightGBM algorithm. Finally, UMAP was utilized to visualize the 2D distribution of the nine clusters, which were identified based on endpoint-specific similarity (Silhouette score of 0.42). All the results and configurations were made available for download in separate files as described in “Report and download of results”section (Additional file 1: Figure S15).

## Conclusion

The chemical grouping workflow was designed to be user-friendly, with a graphical interface that removes the necessity for extensive programming skills, thereby improving its accessibility. It serves as a valuable resource for chemists and researchers seeking to explore and analyze chemical datasets comprehensively. Its ease of use makes it particularly suitable for those new to cheminformatics. For more experienced users, our workflow offers advanced features and flexibility to select different configurations of the grouping process to obtain better results. Furthermore, in-depth customization can be applied in the desktop version of KNIME Analytics Platform, where users can fine-tune the workflow at different stages, tailoring it to their specific needs. This customization capability is invaluable when addressing complex and specialized chemical analysis requirements. The server version of our workflow, integrated with the NIEHS KNIME Server and WebPortal, enhances usability and scalability, making it a powerful tool for collaborative chemical data analysis. It allows multiple users to access and execute the workflow in a streamlined and controlled manner, facilitating teamwork and ensuring reproducibility in research.

Our workflow has implemented all the needed steps for chemical grouping, including data input, molecular descriptor calculation, dimensionality reduction, feature selection, unsupervised clustering, supervised grouping, hyperparameter tuning, and visualization. It provides a comprehensive solution that guides users through these critical steps in the grouping analysis. Moreover, we introduced an interpretation step using SHAP values to identify the most important molecular descriptors contributing to each group and the generated natural language summaries of the explanations aid in understanding the reasons behind the groupings.

Overall, our approach provides a valuable tool for chemists and researchers to explore chemical datasets, gain insights into chemical properties, and facilitate decision-making processes in various applications, including drug discovery, chemical risk assessment, and structure–activity relationship analysis.

### Supplementary Information

Additional file 1. Additional tables and figures of the user interface and functionalities of the workflow.

Additional file 2. SDF file containing the chemical structures of the eye irritation and corrosion dataset.

## Data Availability

The data used in the case study and the chemical grouping workflow are available for download in the KNIME Community Hub at https://hub.knime.com/-/spaces/-/latest/~AnmyNgAW4JMJ_gq4/ and GitHub at https://github.com/NIEHS/Chemical-grouping-workflow. GitHub also provides a tutorial for installation.
