# Supplementary material for: Democratizing cheminformatics: interpretable chemical grouping using an automated KNIME workflow
Source: J Cheminform. 2024 Aug 16;16:101. doi: 10.1186/s13321-024-00894-1 (PMC11330086; doi:10.1186/s13321-024-00894-1)
Supplement: Supplementary file 1 — Additional file 1. Additional tables and figures of the user interface and functionalities of the workflow. [file 13321_2024_894_MOESM1_ESM.docx]

**Support Information**

Democratizing Cheminformatics: Interpretable Chemical Grouping Using an Automated KNIME Workflow

*José T. Moreira-Filho^1^, Dhruv Ranganath^2^, Mike Conway^3^, Charles Schmitt^4^, Nicole Kleinstreuer^1^, and Kamel Mansouri^1*^*

^1^NIH/NIEHS/DTT/NICEATM, RTP, NC, USA; ^2^University of North Carolina at Chapel Hill, NC, USA; ^3^NIH/NIEHS, RTP, NC, USA; ^4^NIH/NIEHS/DTT, RTP, NC, USA.

**Table S1**. Molecular fingerprints.

| **Type of Fingerprint** | **Description** | **Reference** |
| --- | --- | --- |
| Morgan Fingerprints | A type of circular fingerprints generated by Morgan algorithm that encodes the atoms features within a specific diameter through Daylight atomic invariants. | ^1^ |
| FeatMorgan Fingerprints | A variant of the Morgan fingerprints that encode the functional role of the atoms in a chemical structure by a set of pharmacophoric characteristics (e.g., aromatic, halogen, hydrogen-bond acceptor and donor, negatively and positively ionizable). | ^2^ |
| MACCS | A structural key fingerprint where bits are extracted from a list of 166 representative substructures. | ^3^ |

**Table S2.** Hyperparameters and their respective algorithms available for tuning in the chemical grouping workflow using the Optuna method.

| **Algorithm** | **Hyperparameter** | **Brief description** |
| --- | --- | --- |
| K-means | *k number of groups* | The number of clusters to form as well as the number of centroids to generate. |
| K-medoids | *k number of groups* | The number of clusters to form as well as the number of centroids to generate. |
| Hierarchical clustering | Linkage distance (single, complete, or average) | Determines the distance between sets of observations as a function of the pairwise distances between observations. |
|  | Similarity metric (Euclidean distance or Manhattan) | Quantifies how similar or dissimilar two data points are. |
| DBSCAN | ε | The maximum distance between 2 points for them to be considered neighbors of one another. |
|  | *min_samples* | The minimum number of neighbors a point needs to have to be considered a core point. |
| HDBSCAN | *min_cluster_size* | Determines the minimum size of clusters. |
|  | *min_samples* | Defines the number of samples in a neighborhood for a point to be considered a core point. |
|  | *cluster_selection_epsilon* | A distance threshold that sets the minimum distance between clusters. |
|  | *alph*a | Used in the computation of the hierarchical cluster tree, influencing the cluster selection process. Specifically, it relates to the decay rate for the minimum cluster stability score. |
| UMAP | *n_neighbors* | Controls how UMAP balances local versus global structure in the data. |
|  | *min_dist* | Controls how tightly UMAP is allowed to pack points together. |
| t-SNE | *perplexity* | Controls how UMAP balances local versus global structure in the data. |
|  | *learning_rate* | Controls the scale of adjustments made to the positions of points in the low-dimensional space during each iteration of the optimization. |

DBSCAN, Density-Based Spatial Clustering of Applications with Noise; HDBSCAN, Hierarchical Density-Based Spatial Clustering of Applications with Noise; UMAP, Uniform Manifold Approximation and Projection; t-SNE, t-distributed Stochastic Neighbor Embedding; ε, epsilon.

**Table S3.** Interpretation of supervised classification analysis automatically generated by the LLM GPT 3.5 Turbo.

| **Cluster** | **GPT interpretation** |  |
| --- | --- | --- |
| 0 | 1. Explanation of Molecular Descriptors: - ATSC5v: Autocorrelation of lag 5 weighted by van der Waals volumes. It measures the spatial distribution of atoms in a molecule, specifically focusing on the van der Waals volumes of atoms separated by a distance of 5 bonds. - CIC1: Connectivity index chi-1. It represents the topological connectivity of a molecule by counting the number of bonds between pairs of atoms. - ATSC3se: Autocorrelation of lag 3 weighted by Sanderson electronegativities. It quantifies the spatial distribution of atoms in a molecule, taking into account the electronegativities of atoms separated by a distance of 3 bonds. - AATS0s: Average atomic Sanderson electronegativity weighted by atomic surface areas. It characterizes the average electronegativity of atoms in a molecule, considering their surface areas. - ATSC8pe: Autocorrelation of lag 8 weighted by polarizabilities. It measures the spatial distribution of atoms in a molecule, taking into account their polarizabilities.  2. Analysis of Descriptor Values and Cluster Results: To understand why these descriptors were important in grouping compounds in cluster 0, we need to compare the values of these descriptors within each cluster. Cluster 0: - ATSC5v: The values of ATSC5v in cluster 0 are generally positive, indicating a higher spatial distribution of atoms with larger van der Waals volumes. - CIC1: The values of CIC1 in cluster 0 vary, suggesting different levels of topological connectivity among the compounds. - ATSC3se: The values of ATSC3se in cluster 0 are mostly positive, indicating a higher spatial distribution of atoms with larger electronegativities. - AATS0s: The values of AATS0s in cluster 0 are generally positive, suggesting a higher average electronegativity of atoms with larger surface areas. - ATSC8pe: The values of ATSC8pe in cluster 0 are mostly positive, indicating a higher spatial distribution of atoms with larger polarizabilities.  3. Summary of Results: In cluster 0, compounds are grouped based on their spatial distribution of atoms, topological connectivity, electronegativities, average electronegativity of atoms, and polarizabilities. The positive values of ATSC5v, ATSC3se, AATS0s, and ATSC8pe suggest that compounds in cluster 0 have larger van der Waals volumes, larger electronegativities, larger surface areas, and larger polarizabilities, respectively. The varying values of CIC1 indicate different levels of topological connectivity among the compounds. These descriptors collectively contribute to the grouping of compounds in cluster 0, highlighting their structural and chemical similarities. | |
| 1 | 1. Explanation of Molecular Descriptors: - MIC1: This descriptor represents the molecular information content index for the first order adjacency matrix. It quantifies the complexity of the molecular structure based on the connectivity of atoms. – AATSC1i: This descriptor is the average atomic contribution of atomic Sanderson electronegativity for all atoms in the molecule. It provides information about the electronegativity distribution within the molecule. – MIC0: Similar to MIC1, this descriptor represents the molecular information content index for the zeroth order adjacency matrix. It captures the overall complexity of the molecular structure. – ATSC1se: This descriptor is the average topological charge index for all atoms in the molecule. It reflects the charge distribution within the molecule. – CIC4: This descriptor is the fourth order connectivity index. It characterizes the topological connectivity of atoms within the moleculee.  2. Analysis of Descriptor Values and Cluster Results: To understand why these descriptors were important in grouping compounds in cluster 1, we need to compare their values within each cluster. Cluster 1: - MIC1: The values of MIC1 in cluster 1 are generally negative, indicating that the compounds in this cluster have lower structural complexity and connectivity. - AATSC1i: The AATSC1i values in cluster 1 are consistently zero, suggesting that the electronegativity distribution within the compounds is uniform. - MIC0: Similar to MIC1, the MIC0 values in cluster 1 are negative, indicating lower overall structural complexity. - ATSC1se: The ATSC1se values in cluster 1 are generally positive, suggesting a positive charge distribution within the compounds. - CIC4: The CIC4 values in cluster 1 vary, but they are generally close to zero, indicating a moderate level of topological connectivity.  3. Summary of Results: Based on the analysis of the key descriptors in cluster 1, we can conclude that compounds in this cluster have lower structural complexity and connectivity (as indicated by negative MIC1 and MIC0 values), a uniform electronegativity distribution (AATSC1i values of zero), a positive charge distribution (positive ATSC1se values), and moderate topological connectivity (CIC4 values close to zero). These descriptors were important in grouping compounds in cluster 1 because they capture the structural and chemical characteristics that distinguish these compounds from others in the dataset. | |
| 2 | 1. Explanation of Molecular Descriptors: - ATS0i: Average topological shape index of order 0. It represents the average shape of a molecule based on its connectivity. - CIC4: Fourth-order connectivity index. It quantifies the complexity of the molecular structure by considering the number of paths of length 4 between pairs of atoms. - MIC1: First-order molecular connectivity index. It measures the average distance between pairs of atoms in a molecule. - AATS0s: Average atom-type electrotopological state index of order 0. It characterizes the electronic and topological properties of a molecule based on the types of atoms present. - ATS0pe: Average topological shape index of order 0 weighted by atomic polarizabilities. It incorporates the polarizability of atoms into the calculation of the shape index.  2. Analysis of Descriptor Values and Cluster Results: To understand why these descriptors were important in grouping compounds in cluster 2, we need to compare the values of these descriptors within each cluster. Cluster 2: - ATS0i: The average value of ATS0i in cluster 2 is relatively high, indicating that the molecules in this cluster have a more complex and diverse shape compared to other clusters. - CIC4: The CIC4 values in cluster 2 are generally lower, suggesting that the molecular structures in this cluster have fewer complex paths of length 4 between atoms. - MIC1: The MIC1 values in cluster 2 are higher, indicating that the average distance between pairs of atoms in the molecules of this cluster is greater. - AATS0s: The AATS0s values in cluster 2 are relatively high, suggesting that the electronic and topological properties of the molecules in this cluster are distinct from those in other clusters. - ATS0pe: The ATS0pe values in cluster 2 are higher, indicating that the shape of the molecules in this cluster, weighted by atomic polarizabilities, is more diverse.  3. Summary of Results: The molecular descriptors ATS0i, CIC4, MIC1, AATS0s, and ATS0pe were found to be important in grouping compounds in cluster 2. The analysis of these descriptors within cluster 2 revealed that the molecules in this cluster have a more complex and diverse shape, fewer complex paths of length 4 between atoms, greater average distance between pairs of atoms, distinct electronic and topological properties, and a more diverse shape weighted by atomic polarizabilities. These findings suggest that cluster 2 represents a distinct group of compounds with unique structural and chemical characteristics. | |
| 3 | 1. Explanation of Molecular Descriptors: - ATSC5v: Autocorrelation of lag 5 weighted by van der Waals volume. It measures the spatial distribution of atoms in a molecule, specifically focusing on the contribution of atoms with larger van der Waals volumes. - ATSC1se: Autocorrelation of lag 1 weighted by Sanderson electronegativity. It quantifies the similarity between atoms in terms of their electronegativity, with a focus on the contribution of atoms with a smaller electronegativity. - ETA_eta_FL: First order molecular connectivity index weighted by fractional atomic polarizabilities. It characterizes the connectivity between atoms in a molecule, taking into account the polarizability of the atoms. - ATS0i: Autocorrelation of lag 0 weighted by atomic Sanderson electronegativity. It measures the similarity between atoms in terms of their electronegativity, with equal weight given to all atoms. - ATSC3se: Autocorrelation of lag 3 weighted by Sanderson electronegativity. It quantifies the similarity between atoms in terms of their electronegativity, with a focus on the contribution of atoms with a smaller electronegativity.  2. Analysis of Descriptor Values and Cluster Results: To analyze the importance of these descriptors in grouping compounds in cluster 3, we can compare the values of these descriptors within each cluster. Cluster 3: - ATSC5v: The values of ATSC5v in cluster 3 are generally positive, indicating a higher spatial distribution of atoms with larger van der Waals volumes in these compounds. – ATSC1se: The values of ATSC1se in cluster 3 are also positive, suggesting a similarity in electronegativity between atoms in these compounds, with a focus on atoms with smaller electronegativity. – ETA_eta_FL: The values of ETA_eta_FL in cluster 3 are generally negative, indicating a lower first order molecular connectivity index weighted by fractional atomic polarizabilities in these compounds. – ATS0i: The values of ATS0i in cluster 3 are generally positive, suggesting a similarity in electronegativity between atoms in these compounds, with equal weight given to all atoms. – ATSC3se: The values of ATSC3se in cluster 3 are generally negative, indicating a similarity in electronegativity between atoms in these compounds, with a focus on atoms with smaller electronegativity.  3. Summary of Results: Based on the analysis of the molecular descriptors within cluster 3, it can be concluded that compounds in this cluster have a higher spatial distribution of atoms with larger van der Waals volumes, a similarity in electronegativity between atoms (with a focus on atoms with smaller electronegativity), a lower first order molecular connectivity index weighted by fractional atomic polarizabilities, and a similarity in electronegativity between atoms (with equal weight given to all atoms). These descriptors were important in grouping compounds in cluster 3, as they capture key structural and chemical properties that differentiate these compounds from those in other clusters. | |
| 4 | 1. Molecular Descriptors: - CIC1: The first component of the connectivity index (CIC) is a topological descriptor that measures the sum of the squares of the bond lengths in a molecule. It provides information about the size and shape of the molecule. - ATSC5p: Autocorrelation of lag 5 weighted by atomic Sanderson electronegativity. It quantifies the distribution of electronegativity in the molecule. - ATSC3se: Autocorrelation of lag 3 weighted by atomic Sanderson secondary electronegativity. It measures the distribution of secondary electronegativity in the molecule. - ATSC1se: Autocorrelation of lag 1 weighted by atomic Sanderson secondary electronegativity. It provides information about the distribution of secondary electronegativity in the molecule. - MIC0: Moran autocorrelation of lag 0 weighted by atomic Sanderson electronegativity. It quantifies the spatial autocorrelation of electronegativity in the molecule.  2. Analysis of Descriptor Values and Cluster Results: To understand why these descriptors were important in grouping compounds in cluster 4, we need to compare the values of these descriptors within each cluster. Cluster 4: - CIC1: The values of CIC1 in cluster 4 are generally negative, indicating that the molecules in this cluster tend to be smaller in size and have a more compact shape. - ATSC5p: The values of ATSC5p in cluster 4 are mostly negative, suggesting that the distribution of electronegativity in these molecules is more uniform. - ATSC3se: The values of ATSC3se in cluster 4 are also negative, indicating a more uniform distribution of secondary electronegativity in the molecules. - ATSC1se: The values of ATSC1se in cluster 4 are close to zero, suggesting a less pronounced distribution of secondary electronegativity. - MIC0: The values of MIC0 in cluster 4 are mostly positive, indicating a positive spatial autocorrelation of electronegativity in the molecules.  3. Summary of Results: Based on the analysis of the key descriptors in cluster 4, we can conclude that the molecules in this cluster tend to be smaller in size and have a more compact shape. They also exhibit a more uniform distribution of electronegativity and secondary electronegativity. Additionally, there is a positive spatial autocorrelation of electronegativity within this cluster. These findings suggest that the molecules in cluster 4 share similar structural and chemical properties, which are captured by the important descriptors. | |
| 5 | 1. Molecular Descriptors: - ATSC0dv: Autocorrelation of the atomic Sanderson electronegativity weighted by van der Waals volumes. It measures the distribution of electronegativity in a molecule. - AATSC1i: Average atomic Sanderson electronegativity weighted by van der Waals volumes. It represents the average electronegativity of atoms in a molecule. - AATS1are: Average atomic Sanderson polarizability weighted by atomic masses. It indicates the average polarizability of atoms in a molecule. - RotRatio: Ratio of the number of rotatable bonds to the number of heavy atoms in a molecule. It quantifies the flexibility of a molecule. - ATS0i: Autocorrelation of the atomic Sanderson electronegativity weighted by atomic masses. It measures the distribution of electronegativity in a molecule. 2. Analysis of Descriptor Values and Cluster Results: To understand why these descriptors were important in grouping compounds in cluster 5, we need to compare the values of these descriptors within each cluster. Cluster 5: - ATSC0dv: The values of ATSC0dv in cluster 5 are generally positive, indicating a higher distribution of electronegativity in these compounds compared to other clusters. - AATSC1i: The values of AATSC1i in cluster 5 are close to zero, suggesting that the average electronegativity of atoms in these compounds is similar. - AATS1are: The values of AATS1are in cluster 5 are also close to zero, indicating that the average polarizability of atoms in these compounds is similar. - RotRatio: The values of RotRatio in cluster 5 are generally low, implying that the compounds in this cluster have a lower flexibility due to fewer rotatable bonds. - ATS0i: The values of ATS0i in cluster 5 are mostly positive, indicating a higher distribution of electronegativity in these compounds compared to other clusters. 3. Summary of Results: Based on the analysis of the key descriptors within each cluster, it can be concluded that cluster 5 is characterized by compounds with a higher distribution of electronegativity, similar average electronegativity and polarizability, lower flexibility, and higher distribution of electronegativity. These descriptors were important in grouping compounds in cluster 5 as they capture the similarities in electronegativity, polarizability, and flexibility among the compounds. | |
| 6 | 1. Molecular Descriptors: - ATSC1se: Autocorrelation of lag 1 weighted by atomic Sanderson electronegativity. - ATS7dv: Autocorrelation of lag 7 weighted by atomic van der Waals volume. - ATSC3se: Autocorrelation of lag 3 weighted by atomic Sanderson electronegativity. - CIC2: Connectivity index chi 2. - CIC1: Connectivity index chi 1.  2. Analysis of Descriptor Values and Cluster Results: To understand why these descriptors were important in grouping compounds in cluster 6, we need to compare the values of these descriptors within each cluster. Cluster 6: - ATSC1se: The values of ATSC1se in cluster 6 are generally positive, indicating a higher autocorrelation of lag 1 weighted by atomic Sanderson electronegativity. This suggests that compounds in cluster 6 have similar electronegativity patterns in their molecular structures. - ATS7dv: The values of ATS7dv in cluster 6 are mostly negative, indicating a lower autocorrelation of lag 7 weighted by atomic van der Waals volume. This suggests that compounds in cluster 6 have diverse van der Waals volumes in their molecular structures. - ATSC3se: The values of ATSC3se in cluster 6 are also positive, indicating a higher autocorrelation of lag 3 weighted by atomic Sanderson electronegativity. This further supports the similarity in electronegativity patterns among compounds in cluster 6. - CIC2: The values of CIC2 in cluster 6 are generally negative, indicating a lower connectivity index chi 2. This suggests that compounds in cluster 6 have less complex molecular connectivity patterns. - CIC1: The values of CIC1 in cluster 6 are mostly negative, indicating a lower connectivity index chi 1. This further supports the less complex molecular connectivity patterns observed in compounds in cluster 6.  3. Summary of Results: The analysis of the key descriptors ['ATSC1se', 'ATS7dv', 'ATSC3se', 'CIC2', 'CIC1'] within cluster 6 reveals that compounds in this cluster have similar electronegativity patterns (ATSC1se and ATSC3se) but diverse van der Waals volumes (ATS7dv). Additionally, compounds in cluster 6 have less complex molecular connectivity patterns (lower values of CIC2 and CIC1). These findings suggest that the compounds in cluster 6 share common structural features related to electronegativity and molecular connectivity, but exhibit variability in van der Waals volumes. | |
| 7 | 1. Explanation of Molecular Descriptors: - CIC1: The first component of the connectivity index (CIC) is a topological descriptor that measures the sum of the squares of the bond lengths in a molecule. It provides information about the size and shape of the molecule. - ATSC5p: Autocorrelation of lag 5 weighted by atomic Sanderson electronegativity (ATS) is a topological descriptor that quantifies the electronegativity distribution in a molecule. It provides information about the polarity and charge distribution. - AATSC1i: Average atomic Sanderson electronegativity of 1st order neighbors weighted by atomic Sanderson electronegativity (AATSC) is a topological descriptor that characterizes the electronegativity of the atoms in a molecule and their surrounding environment. - ATS7are: Autocorrelation of lag 7 weighted by atomic Sanderson polarizability (ATS) is a topological descriptor that captures the polarizability distribution in a molecule. It provides information about the flexibility and response to external electric fields. - ATSC2are: Autocorrelation of lag 2 weighted by atomic Sanderson polarizability (ATS) is a topological descriptor that measures the polarizability distribution in a molecule. It provides information about the ability of the molecule to undergo induced dipole moments.  2. Analysis of Descriptor Values and Cluster Results: To understand why these descriptors were important in grouping compounds in cluster 7, we can compare the values of these descriptors within each cluster. - Cluster 7: The compounds in this cluster have high values of CIC1, ATSC5p, AATSC1i, ATS7are, and ATSC2are. This suggests that the compounds in this cluster are likely to have larger sizes, higher electronegativity, higher polarizability, and more flexible structures compared to compounds in other clusters. By analyzing the values of these descriptors within each cluster, we can observe distinct patterns that contribute to the importance of these descriptors in grouping compounds in cluster 7. The high values of CIC1 indicate that the compounds in cluster 7 have larger and more complex structures compared to other clusters. The high values of ATSC5p and AATSC1i suggest that the compounds in cluster 7 have higher electronegativity and a more polarized distribution of charges. The high values of ATS7are and ATSC2are indicate that the compounds in cluster 7 have higher polarizability and are more flexible in response to external electric fields.  3. Summary of Results: The analysis of the molecular descriptors revealed that compounds in cluster 7 have larger sizes, higher electronegativity, higher polarizability, and more flexible structures compared to compounds in other clusters. The descriptors CIC1, ATSC5p, AATSC1i, ATS7are, and ATSC2are were found to be important in distinguishing compounds in cluster 7. These descriptors provide information about the size, electronegativity, charge distribution, polarizability, and flexibility of the compounds. The findings suggest that compounds in cluster 7 may have unique structural and chemical properties that differentiate them from compounds in other clusters. | |


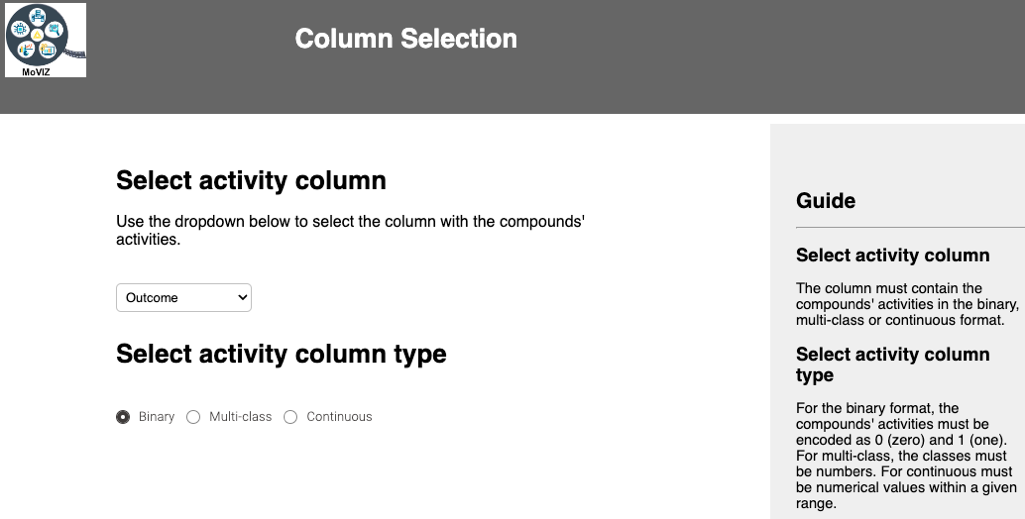


**Figure S1**. Select column page.


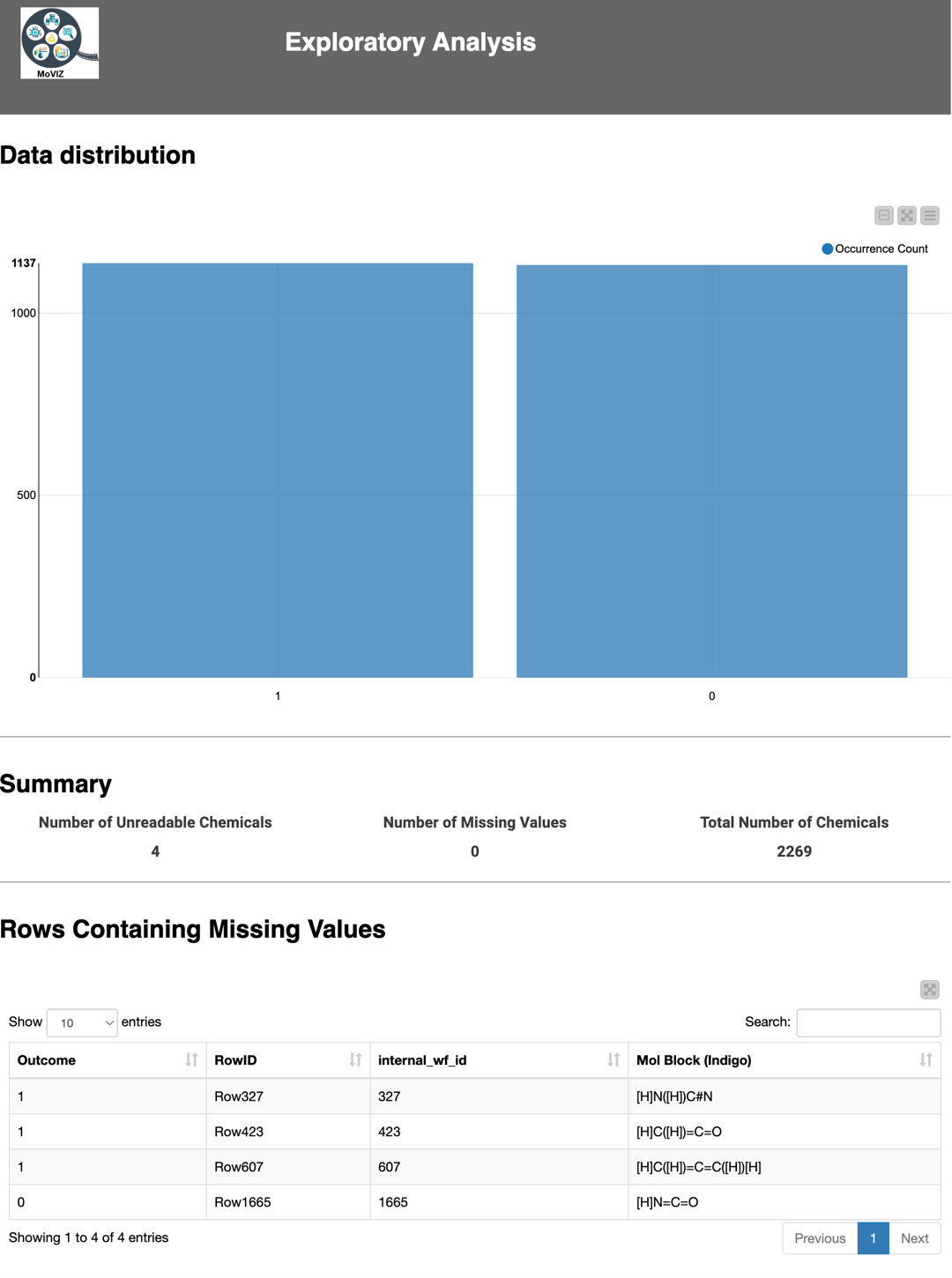


**Figure S2**. Exploratory data analysis.


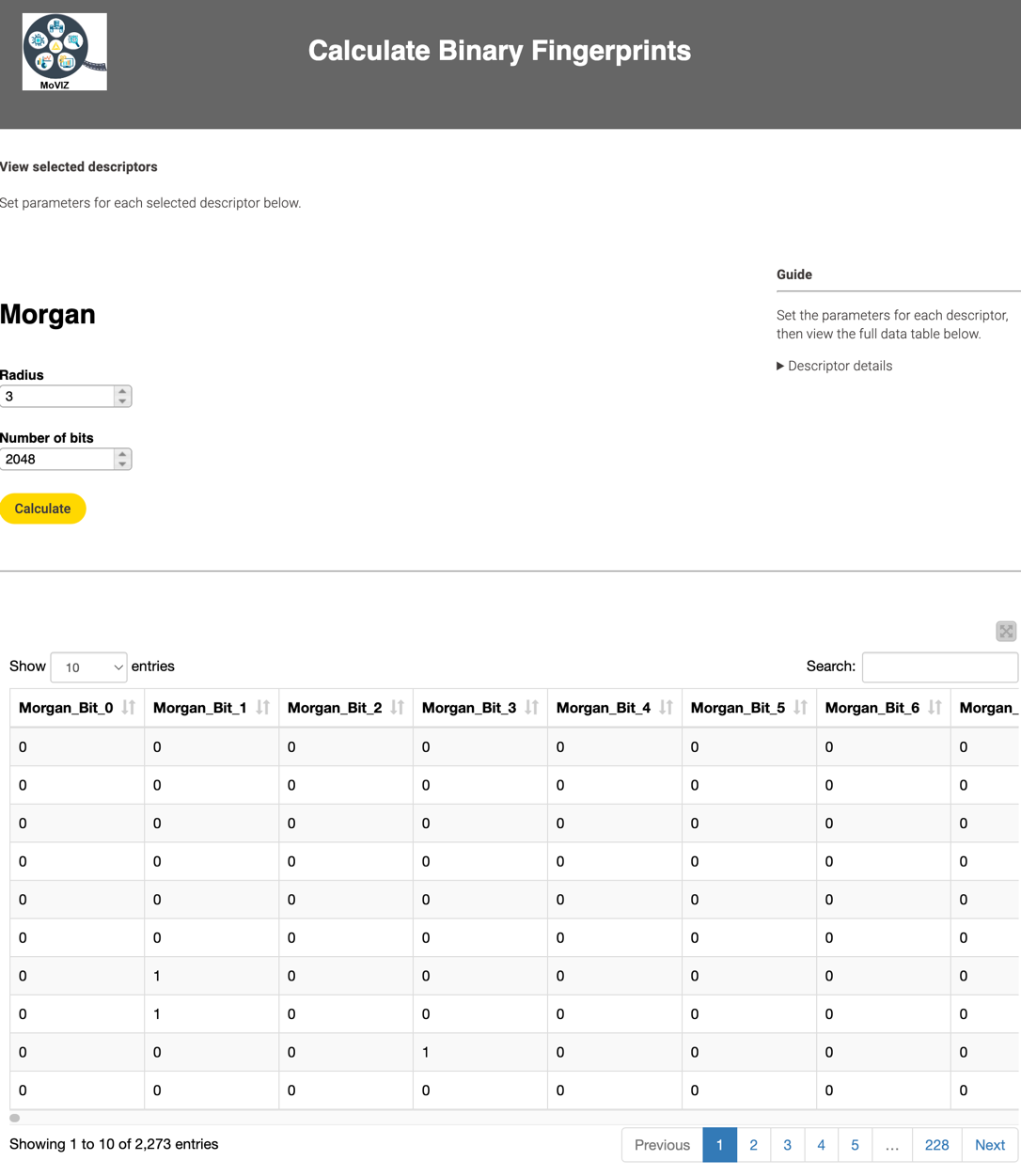


**Figure S3**. Morgan fingerprints configuration and visualization.


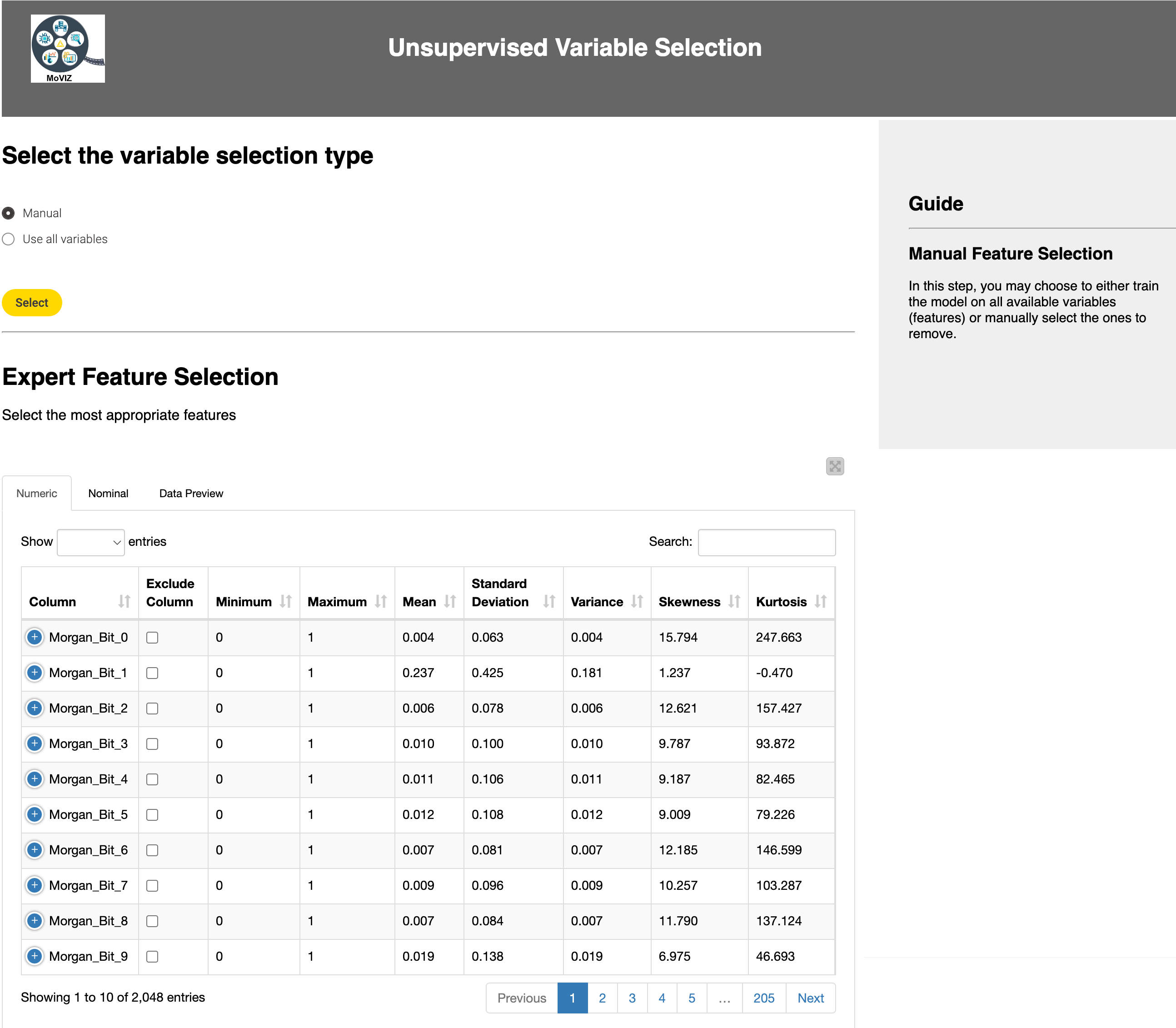


**Figure S4.** Page for manual feature selection.

**
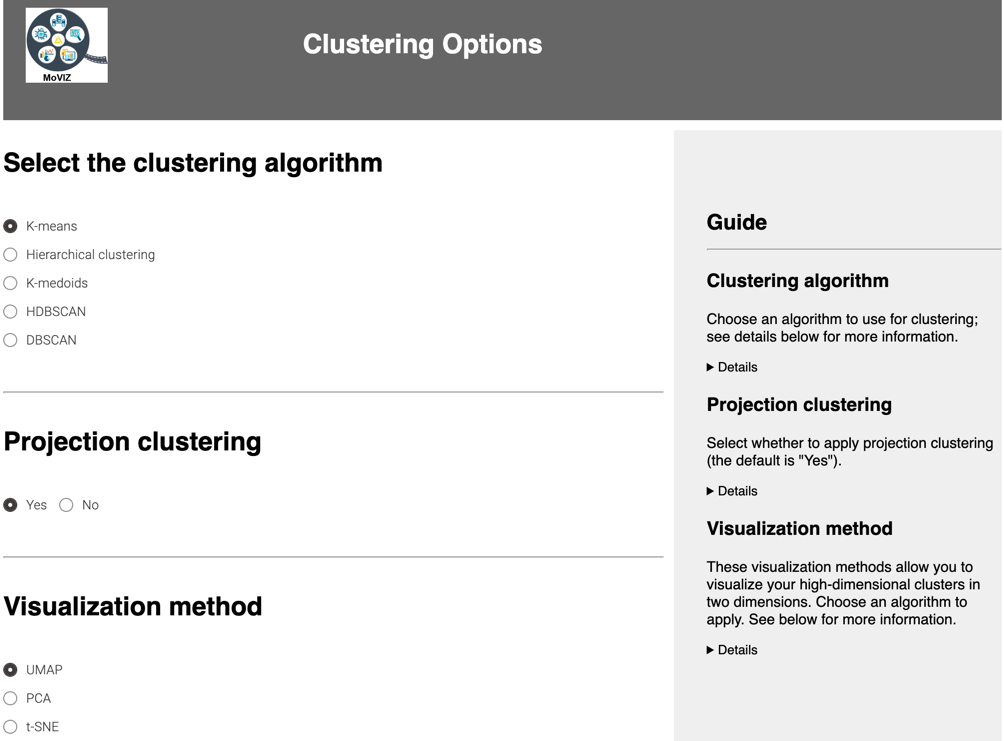
**

**Figure S5**. Chemical grouping configuration page.

**
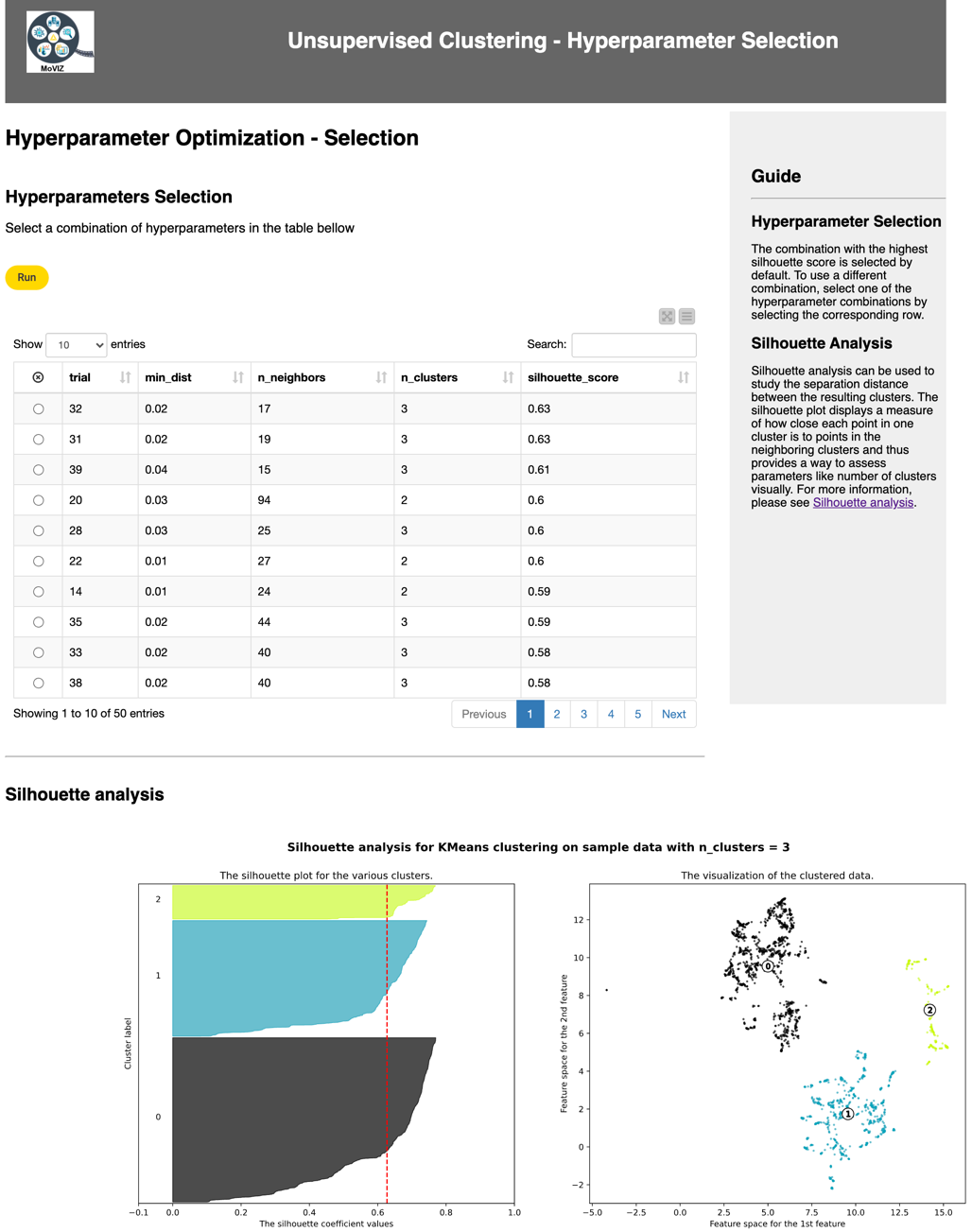
**

**Figure S6.** Hyperparameter selection page and Silhouette analysis of the unsupervised clustering of the case study.


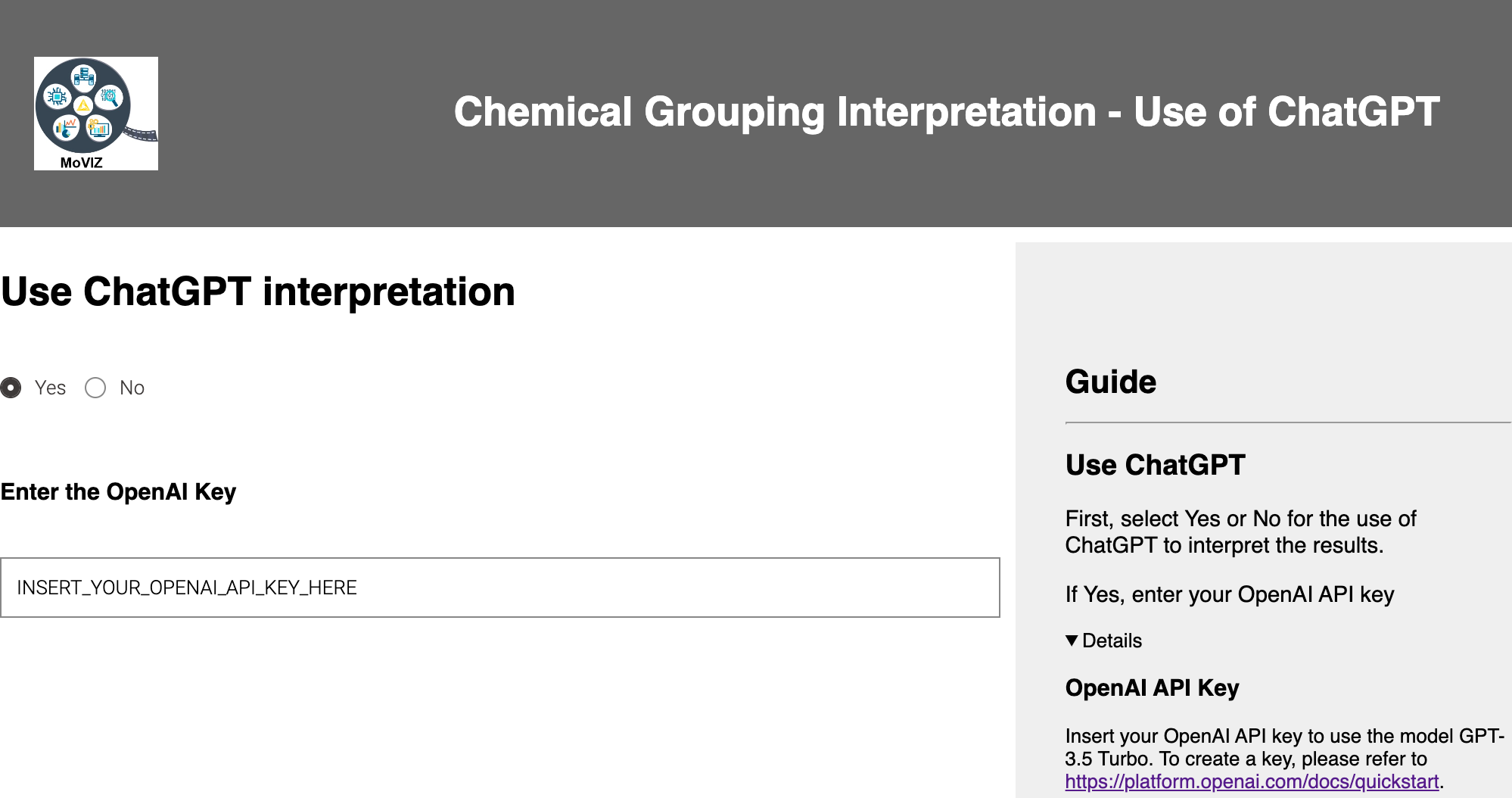


**Figure S7.** Selection of LLM GPT-3.5 Turbo for interpretation of the grouping results and insetion of the user’s OpenAI API Key.

**
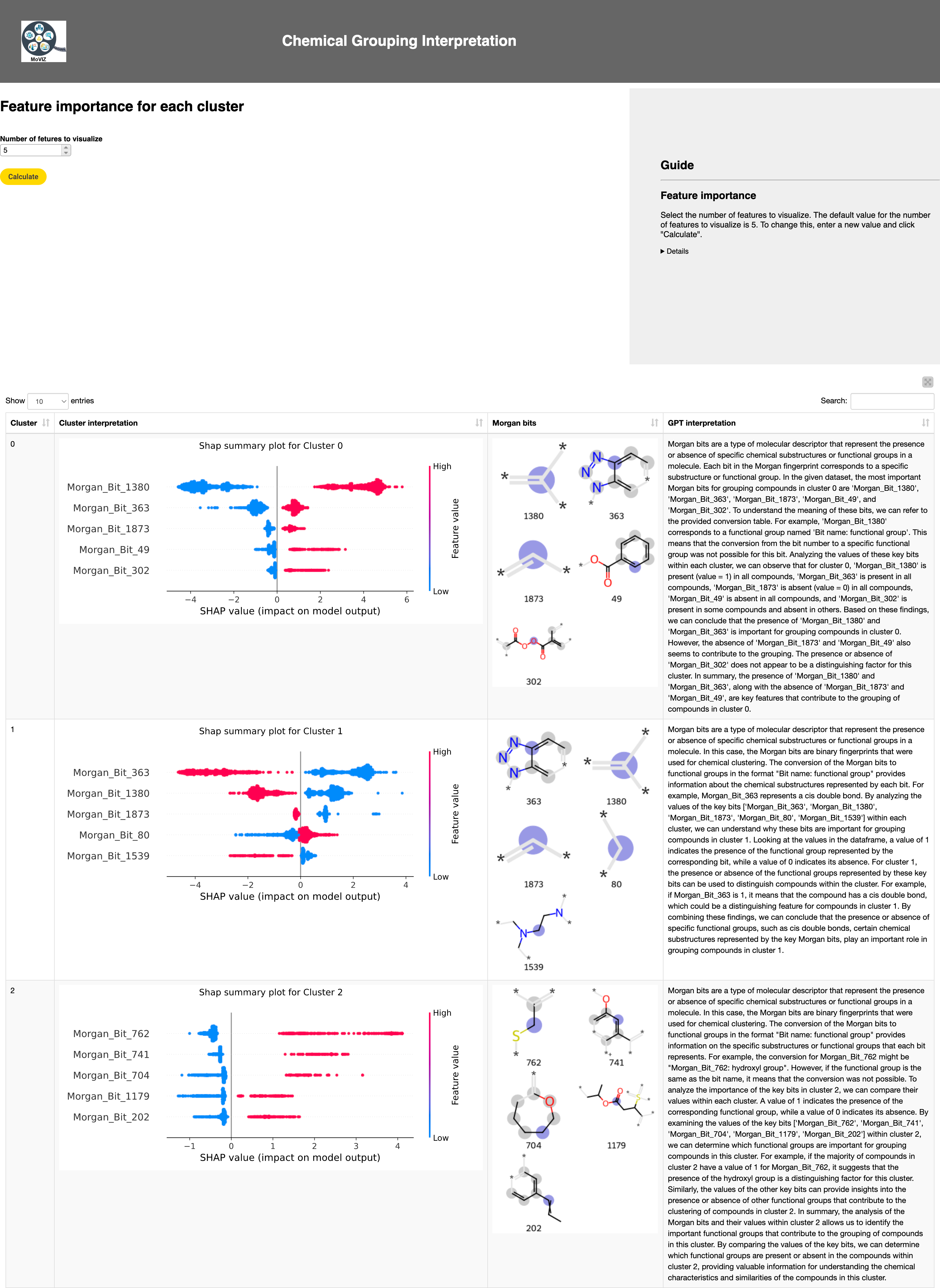
**

**Figure S8.** Complete chemical grouping interpretation of the unsupervised clustering analysis.


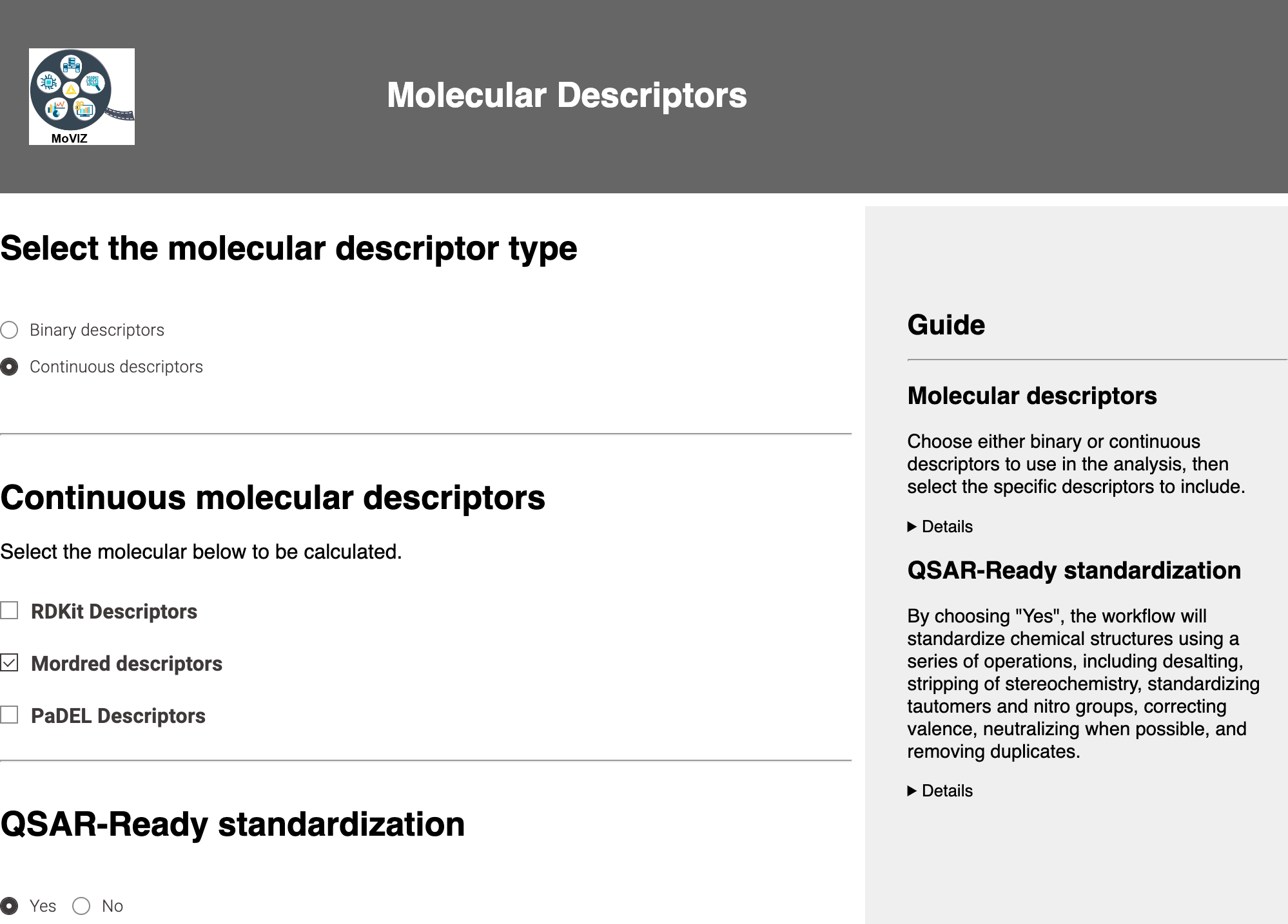


**Figure S9**. Page for the molecular descriptor selection and chemical structure standardization of the supervised classification analysis.


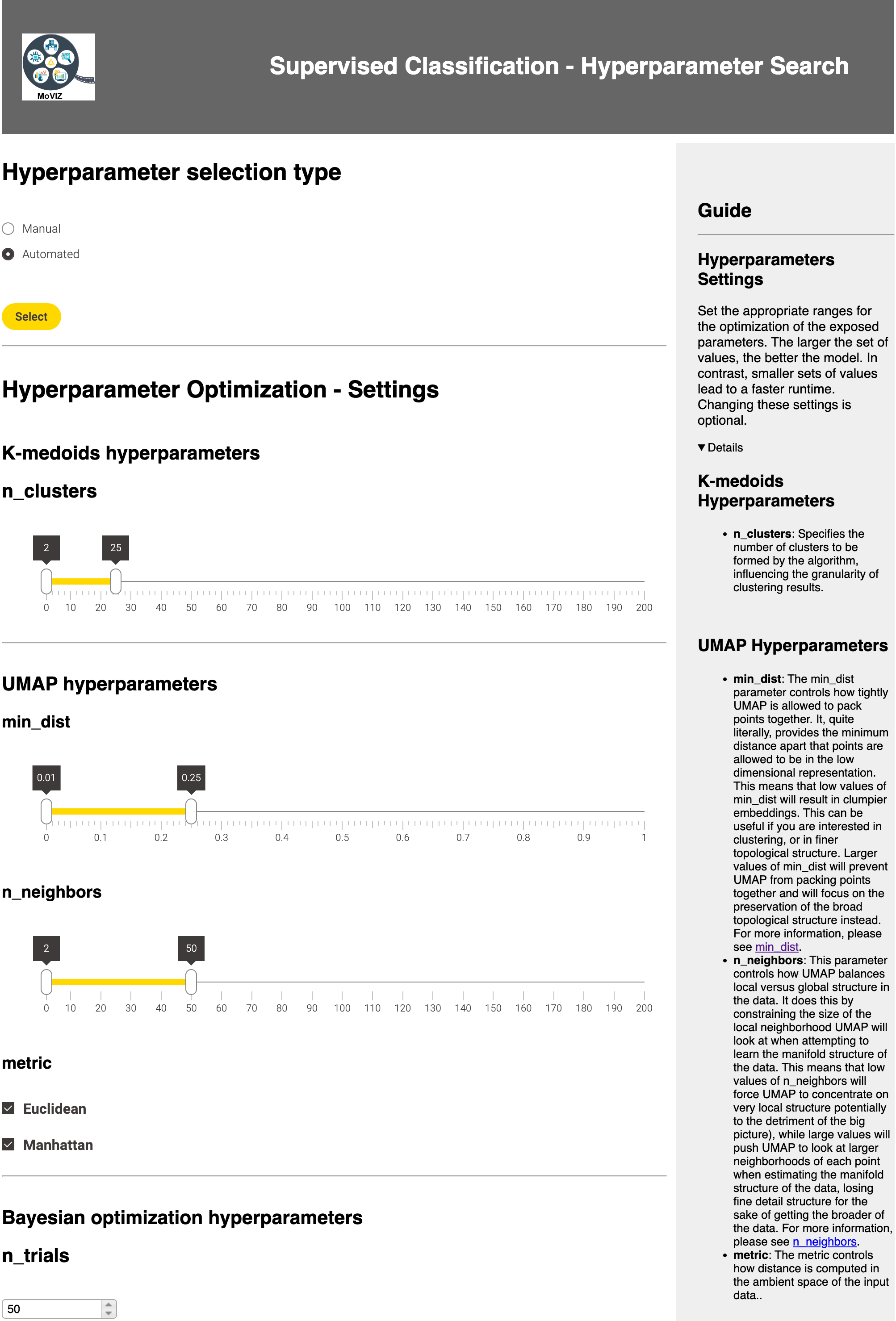


**Figure S10.** Hyperparameter tuning page of the K-medoids clustering algorithm in the supervised classification analysis. The displayed hyperparameters’ ranges were used in the case study.


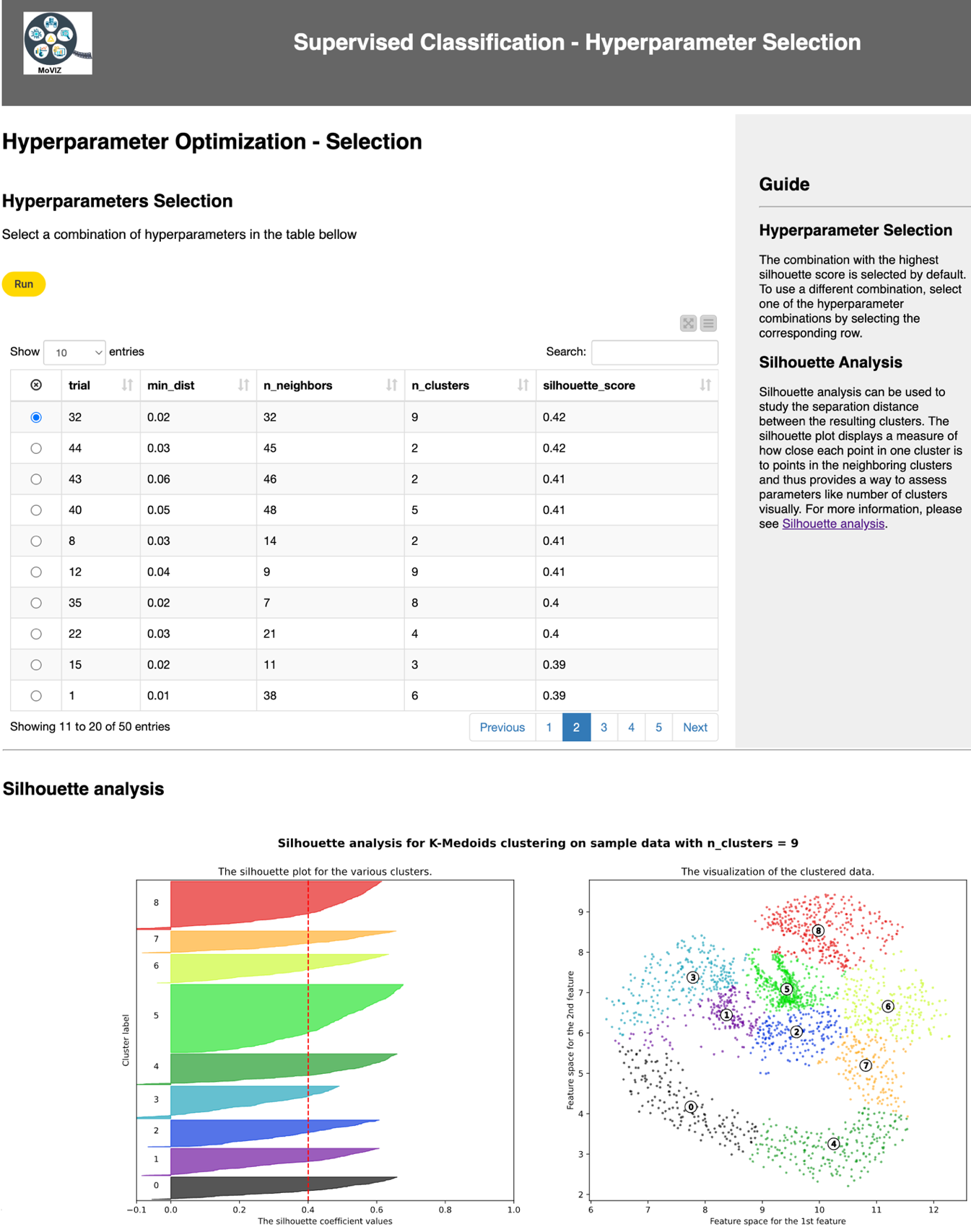


**Figure S11.** Hyperparameter selection page of the supervised classification analysis and the Silhouette analysis.

**
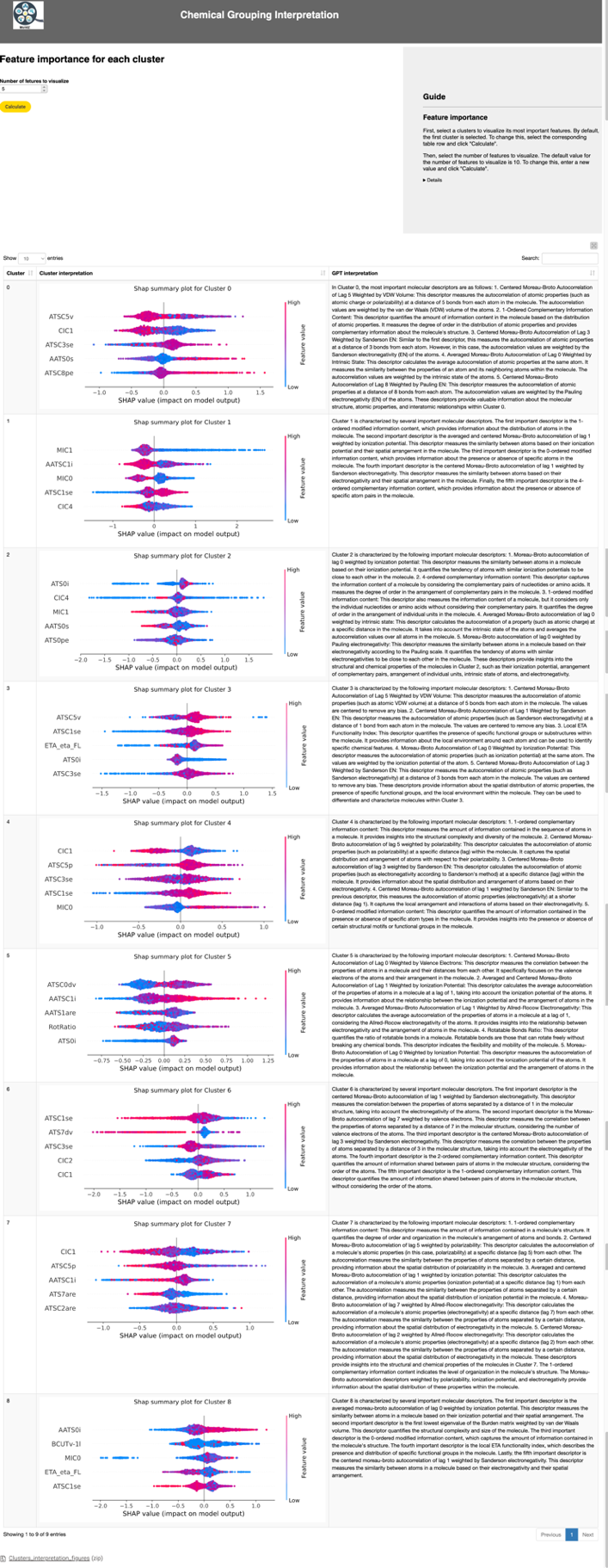
**

**Figure S12.** Complete chemical grouping interpretation of the supervised classification analysis.


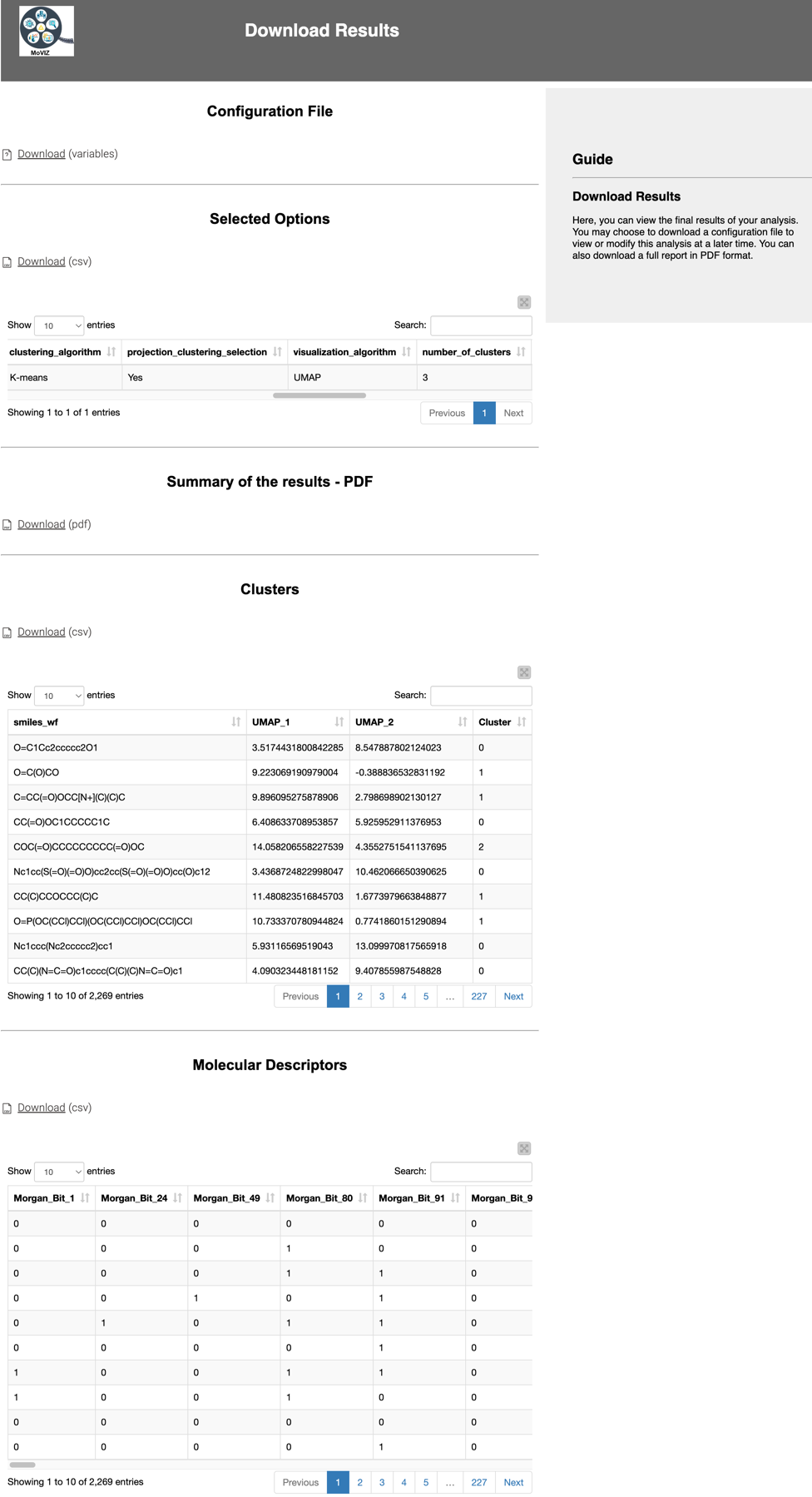


**Figure S13.** Report and results download page of the unsupervised clustering analysis.

**
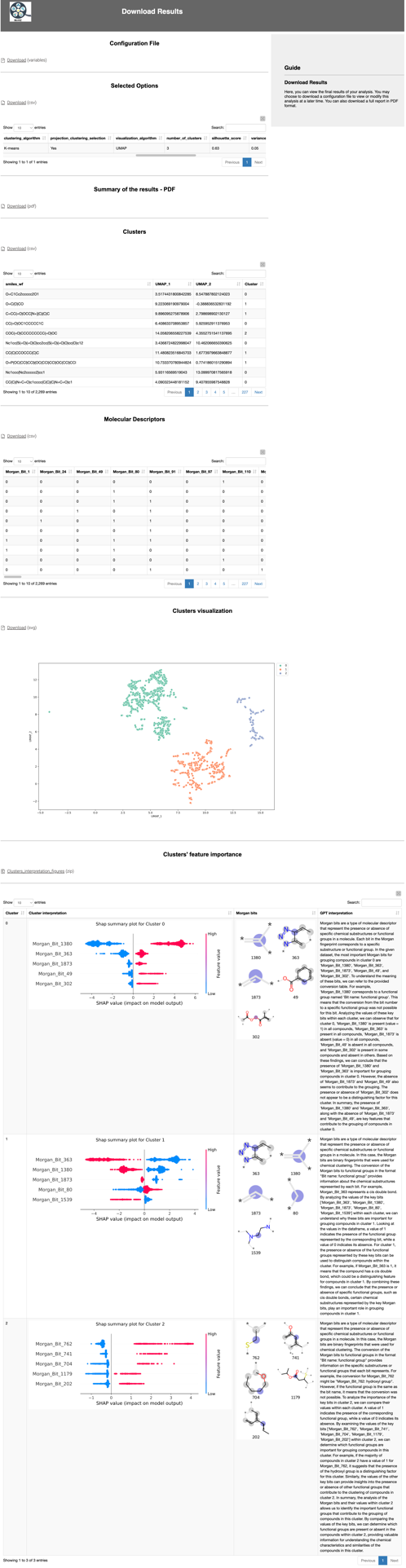
**

**Figure S14.** Complete report and results download page of the unsupervised clustering analysis.


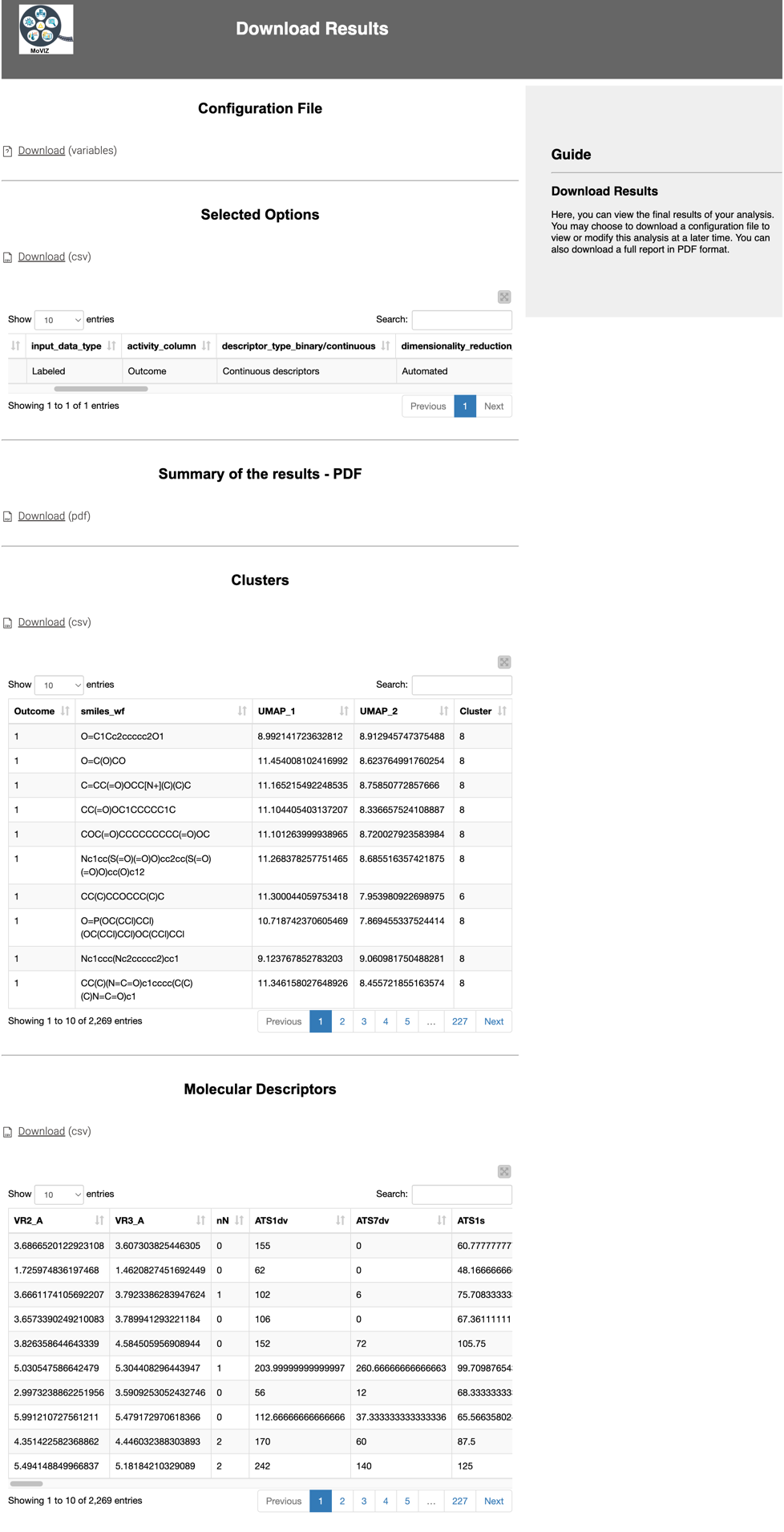


**Figure S15.** Report and results download page of the supervised classification analysis.

Bibliography

(1) Rogers, D.; Hahn, M. Extended-Connectivity Fingerprints. *J. Chem. Inf. Model.* **2010**, *50* (5), 742–754. <https://doi.org/10.1021/ci100050t>.

(2) Cereto-Massagué, A.; Ojeda, M. J.; Valls, C.; Mulero, M.; Garcia-Vallvé, S.; Pujadas, G. Molecular Fingerprint Similarity Search in Virtual Screening. *Methods* **2015**, *71*, 58–63. <https://doi.org/10.1016/j.ymeth.2014.08.005>.

(3) Durant, J. L.; Leland, B. A.; Henry, D. R.; Nourse, J. G. Reoptimization of MDL Keys for Use in Drug Discovery. *J. Chem. Inf. Comput. Sci.* **2002**, *42* (6), 1273–1280. <https://doi.org/10.1021/ci010132r>.
